# Individual Patterns of Heart Rate Variability During Prolonged Cognitive Load with Mental Fatigue Induction

**DOI:** 10.3390/life16091553

**Published:** 2026-09-16

**Authors:** Ekaterina I. Borovkova, Anton R. Kiselev, Vladimir A. Shvartz, Mikhail D. Prokhorov, Aleksey N. Hramkov, Anna M. Vakhlaeva, Sergey E. Burmistrov, Ekaterina M. Zinchenko, Svetlana V. Frolova, Ekaterina A. Lazunina, Elena N. Shvartz, Aleksandr V. Kurbako, Boris P. Bezruchko, Anatoly S. Karavaev

**Affiliations:** 1 Institute of Physics, Saratov State University, Saratov 410012, Russia; rubanei@mail.ru (E.I.B.);; 2 Coordinating Center for Fundamental Research, National Medical Research Center for Therapy and Preventive Medicine, Moscow 101990, Russia; 3 Department of Surgical Treatment for Interactive Pathology, Bakulev National Medical Research Center for Cardiovascular Surgery, Moscow 121552, Russia; 4 Kotelnikov Institute of Radio Engineering and Electronics of Russian Academy of Sciences, Saratov Branch, Saratov 410019, Russia; 5 Faculty of Psychology, Saratov State University, Saratov 410012, Russia; odonata1108@yandex.ru (E.M.Z.);

**Keywords:** heart rate variability, mentally fatiguing tasks, Serial Subtraction Task, Working Memory Task, phases of cognitive performance, adaptation phase, optimal performance phase, fatigue phase, index of efficiency of mental performance

## Abstract

We investigated individual dynamics of cognitive performance and heart rate variability (HRV) measures during prolonged mental load. The aim of this study was to identify characteristic phases of cognitive performance and individual patterns of physiological responses induced by prolonged mentally fatiguing tasks. In prolonged experiments with two cognitive tasks of different structure, cognitive performance, HRV measures, and subjective fatigue were analyzed. The proposed integrated index of relative performance made it possible to identify individual phases of cognitive performance: the adaptation phase (AP), the optimal performance phase (OP), and the fatigue phase (FP). The OP was observed in all subjects, the FP in most subjects, whereas the AP was observed in fewer than half of the subjects, indicating pronounced inter-individual variability in cognitive performance dynamics. Two patterns of the initial heart rate (HR) response were identified, ΔHR ≥ 0 and ΔHR < 0, which were associated with differences in the subsequent dynamics of HR and individual HRV measures. The nature of these differences depended on the phase of cognitive performance and the type of task. Among subjects who performed both tasks, approximately half showed the same direction of the initial HR response in the Serial Subtraction Task (SST) and the Working Memory Task (WMT), whereas the remaining subjects showed different directions, indicating limited reproducibility across tasks. The obtained results demonstrate pronounced inter-individual variability in cognitive performance and physiological responses during prolonged mental load and support the need to account for individual dynamics when developing personalized approaches to monitoring mental fatigue.

## 1. Introduction

According to recent estimates [1], workers engaged in mental work represent about half of the global workforce and more than 70% of the workforce in developed countries. Mental fatigue is a key factor that reduces work performance in this group. This state also has a negative effect on students’ learning performance and poses a direct safety risk for operators of complex machinery, air traffic controllers and drivers [2]. Early detection of mental fatigue is therefore an important scientific task.

The level of mental fatigue is traditionally assessed by psychologists using self-report questionnaires and rating scales [3,4]. However, this subjective approach does not allow for continuous real-time monitoring of mental fatigue. A particularly promising practical approach is to assess the level of mental fatigue using biological signals that can be non-invasively recorded from the human body. This approach opens the possibility of creating wearable monitoring devices that can improve the efficiency of people engaged in mental work, optimize individual educational trajectories for students, and enhance the safety of drivers and operators.

Electroencephalography is the main electrophysiological method for objective assessment of mental fatigue during prolonged mentally fatiguing tasks [5,6,7,8,9,10]. Reliable electroencephalogram (EEG) markers of mental fatigue include increased theta- and alpha- components, a decrease in individual frequency of alpha peak, and an increase in fatigue index [5,6,7,8,9,10]. These changes indicate reduced speed and coherence of neural network activity and are associated with impaired cognitive functions, as shown in multiple studies. However, high inter-individual variability and the complexity of automating EEG analysis limit its suitability for large-scale fatigue monitoring. This limitation drives the search for alternative heart rate variability (HRV) measures that can serve as physiological markers of cardiovascular responses to mental load and can be recorded using ergonomic devices [5,6,7,8,9,10].

Particularly promising prospects arise if mental fatigue can be reliably diagnosed from cardiovascular signals, in particular from the electrocardiogram (ECG). Such recorders are inexpensive, ergonomic, and already widely implemented in consumer wearable devices such as smartwatches [11]. ECG signals make it possible to derive heart rate (HR) and HRV measures that characterize cardiovascular regulation and beat-to-beat variability. These measures are influenced, among other factors, by autonomic modulation of cardiac function and can therefore reflect physiological responses associated with changes in cognitive load and the development of mental fatigue [12,13,14,15,16,17].

Systematic reviews and experimental studies have demonstrated complex dynamics of HRV measures during mentally fatiguing tasks [18,19,20,21,22,23]. According to a recent systematic review [19], the most consistent response to prolonged mentally fatiguing tasks is an overall increase in HRV, with the low-frequency (LF) component and RMSSD showing the most robust upward trends. By contrast, changes in widely used measures such as the high-frequency (HF) component and the LF/HF ratio are inconsistent across studies.

However, an analysis of the literature reveals several methodological gaps. First, only a few studies employ experimental protocols lasting more than 3 h, even though prolonged experiments are necessary to model the development of mental fatigue during the working day. Second, HRV measures are rarely evaluated separately across the characteristic phases of cognitive performance, such as the adaptation phase, the optimal performance phase, and the fatigue phase.

In the vast majority of studies, only the values of the indices at the beginning and at the end of task performance are compared. Previous studies analyzed mean group responses, whereas inter-individual variability in cardiovascular responses to cognitive load was largely ignored. However, cardiovascular responses to cognitive load may differ substantially between individuals. As a result, averaging data at the group level may obscure different individual directions of physiological changes and make it difficult to identify patterns associated with the development of mental fatigue.

We hypothesized that HRV dynamics during prolonged cognitive load depend on the phase of cognitive performance, exhibit pronounced inter-individual variability, and differ between cognitive tasks.

Therefore, the aim of this study was to investigate individual dynamics of cognitive performance and HRV measures during different phases of prolonged mental activity while performing two cognitive tasks.

Further development of this research direction may contribute to the development of wearable systems for personalized monitoring of mental fatigue.

## 2. Experimental Protocols

**Figure 1:**
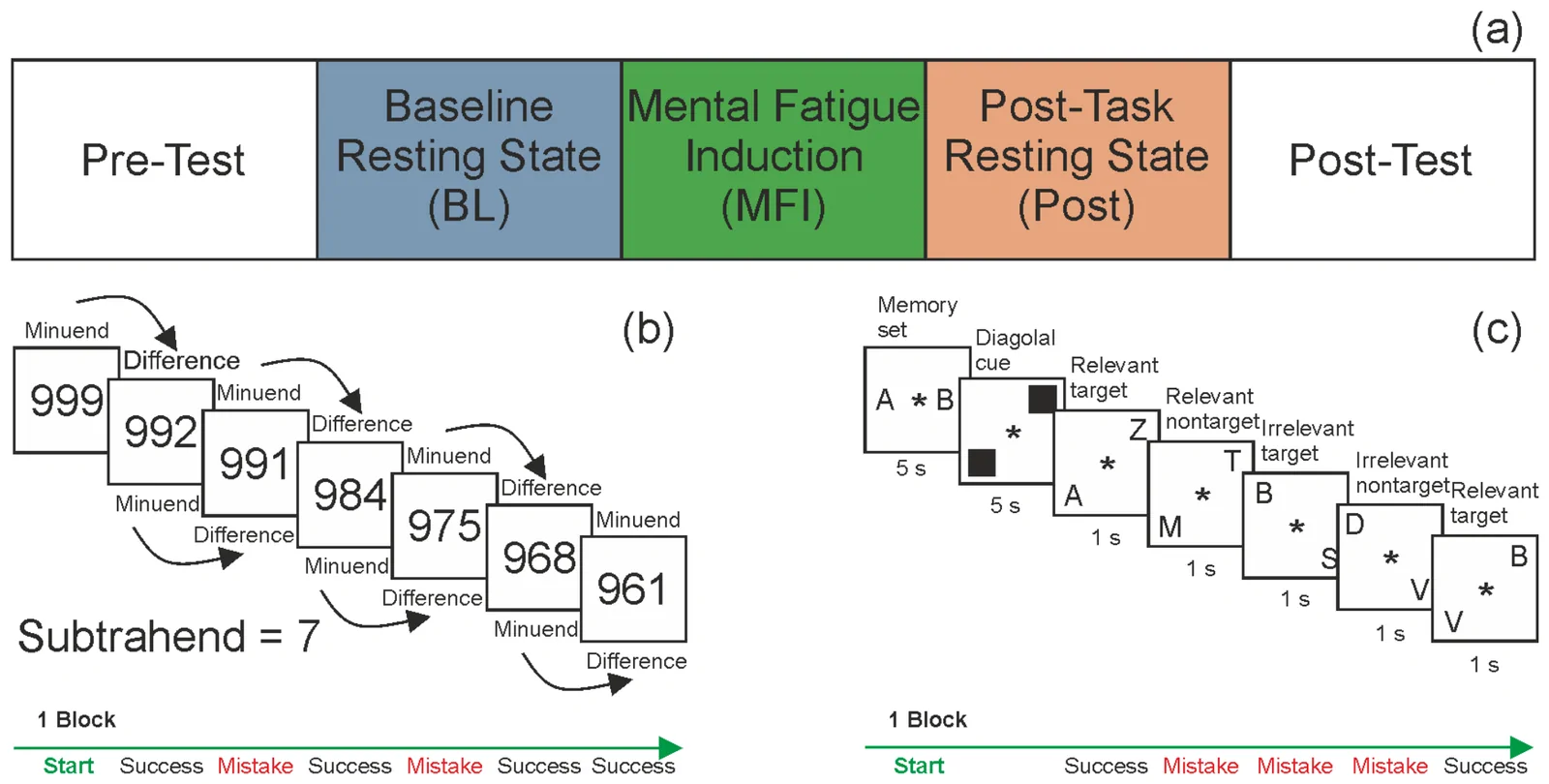
Experimental design. (**a**) Overall structure of the protocol: Pre-Test, BL, MFI, Post, Post-Test. (**b**) SST. (**c**) WMT. The asterisk (*) denotes the fixation point displayed at the center of the screen.

The experimental study was designed according to a five-stage protocol (Figure 1a). In the first stage (Pre-Test), baseline psychological assessment was performed, including the Subjective Mental Fatigue Questionnaire (SMFQ) [24] and the Well-Being, Activity and Mood (WAM) scale [25]. This was followed by a 15 min recording of physiological signals during the Baseline Resting State (BL). The third stage was devoted to Mental Fatigue Induction (MFI) using an individually calibrated prolonged mentally fatiguing task. Immediately after this mental load, a repeated 15 min recording of biosignals was performed (Post-Task Resting State, Post). The final phase of the protocol was a repeated psychological assessment (Post-Test) using the same instruments as in the Pre-Test. The protocol used here follows the commonly adopted design of psychophysiological studies aimed at assessing changes in psychophysiological state before and after prolonged mentally fatiguing tasks [26,27,28,29,30,31,32].

The MFI stage consisted of a series of 15 min blocks of mentally fatiguing tasks. The number of blocks was determined individually for each subject based on their subjective decision to stop and ranged from 4 to 16, with 16 being the maximum number of blocks after which the experiment was stopped by the operator. This resulted in a total duration of prolonged mentally fatiguing tasks from 60 to 240 min. After each block, subjective fatigue was assessed using a Visual Analogue Scale for fatigue (VAS), on which subjects rated their state on a numerical scale from 1 (“no fatigue at all”) to 10 (“complete exhaustion”).

Two different cognitive protocols were used to induce mental fatigue. The Serial Subtraction Task (SST) (Figure 1b) was performed by 45 subjects. In each block, subjects started by subtracting 7 from a given three-digit number and then continued to serially subtract 7 from each subsequent result. When the current value dropped to a two-digit number, a new three-digit number was automatically presented. Responses were entered using a keyboard, without visual feedback on intermediate results or on the accuracy of their responses. This protocol is a modified version of the Paced Auditory Serial Addition Test, adapted for visual stimulus presentation and widely used to induce mental fatigue in psychophysiological studies [33].

The Working Memory Task (WMT) (Figure 1c) was performed by 38 subjects. At the beginning of each block, two target symbols (memory set) and two target screen positions (diagonal cue) were presented for 5 s. Subsequently, various symbols appeared at different positions at a rate of 1 Hz. The subject’s task was to press a key whenever both the currently presented symbol and its position matched one of the memorised targets. The probability of a target stimulus appearing was 1 in 4. This protocol is an adapted version of the *n*-back task designed to assess and induce working-memory-related fatigue [34].

The experiments were conducted in a separate quiet room (6 × 3 m) under constant artificial lighting and at a comfortable air temperature of 22–24 °C. During the recordings, the subjects remained seated in a comfortable medical chair in front of a monitor and performed the cognitive task. The experimenter monitored the experiment from another room. All experiments were conducted in the morning. For 24 h before the study, the subjects abstained from caffeine and alcohol consumption, did not smoke before the experiment, and avoided intense physical activity. Before the experiment, the subjects were allowed to consume food and water up to 2 h before the start of the experiment and to use the toilet. During task performance, food intake and toilet visits were not allowed, whereas water consumption was permitted.

## 3. Experimental Data

The study included 48 healthy subjects (22 men and 26 women) aged 20 (19; 22) years, with data presented as the median and interquartile range. The subjects were university students in their first to fifth years of study. Students were recruited as volunteers through an advertisement. No financial compensation was provided for participation in the experiment. Eligibility criteria required no history of cardiovascular, psychiatric, neurological, or endocrine disorders and no use of psychoactive substances.

A cross-over design was used to compare the two types of prolonged mentally fatiguing tasks. Of the 48 subjects, 45 completed the SST protocol and 38 completed the WMT protocol, with 35 subjects taking part in both tasks. These design features, including the partially overlapping samples, were taken into account in the statistical analysis.

The order of the tasks was randomized. The interval between the two experiments was 4–5 weeks to reduce the possible influence of learning and carryover effects between the tasks.

Throughout the entire experiment, a single-channel ECG was recorded in standard lead I using a certified digital polygraph Encephalan_EEGR-19/26 (Medicom MTD, Taganrog, Russia), with a sampling frequency of 250 Hz and a band-pass filter of 0.016–70 Hz [35].

## 4. Methods

### 4.1. Behavioral Data Analysis

To quantify task performance, two key behavioral measures were recorded in each *i*-th block: standardized accuracy $Z_{i}^{A}$ and standardized reaction time $Z_{i}^{\mathrm{RT}}$. In the SST, accuracy was defined as the proportion of correct arithmetic operations relative to the total number of attempts within a block. Reaction time was assessed as the median time required to perform a single operation (the interval between consecutive responses), expressed in seconds. In the WMT, accuracy was calculated as the proportion of correct responses to target stimuli. Reaction time was defined as the median time in seconds between the presentation of a target stimulus and the button press.

To reduce inter-individual variability, the behavioral measures for each subject were standardized relative to their mean values across all task blocks. We calculated the standardized accuracy in the *i*-th block $Z_{i}^{A}$ and the standardized reaction time in the *i*-th block $Z_{i}^{\mathrm{RT}}$:

(1) $$Z_{i}^{A}=\frac{A_{i}-\mu_{A}}{\sigma_{A}},$$

(2) $$Z_{i}^{\mathrm{RT}}=\frac{{\mathrm{RT}}_{i}-\mu_{\mathrm{RT}}}{\sigma_{\mathrm{RT}}},$$

where $A_{i}$ and ${\mathrm{RT}}_{i}$ are the subject’s accuracy and reaction time in the *i*-th block, respectively, $\mu_{A}$ and $\mu_{\mathrm{RT}}$ are the subject’s mean accuracy and reaction time across all blocks, respectively, and $\sigma_{A}$ and $\sigma_{\mathrm{RT}}$ are the standard deviation of the subject’s accuracy and reaction time across all blocks, respectively.

To obtain an integrated measure of task performance that accounts for the speed–accuracy trade-off, an index of efficiency of mental performance (IES) was calculated for each *i*-th block:

(3) $${\mathrm{IES}}_{i}=\langle Z_{i}^{A}-Z_{i}^{\mathrm{RT}}\rangle ,$$

where 〈 〉 denotes the index averaged within a sliding window of 3 blocks to reduce stochastic fluctuations. A positive value of this index ${\mathrm{IES}}_{i}>0$ indicates performance above the individual mean, whereas a negative value ${\mathrm{IES}}_{i}<0$ indicates performance below the individual mean. This index is similar to the metric used in previous studies [36,37].

### 4.2. Heart Rate Variability Measures

ECG recordings were preprocessed in two stages. In the first stage, R-peaks were detected automatically. An R-peak detection algorithm based on analysis of the second derivative of the ECG signal was used. The original ECG signal was approximated using a second-order smoothing polynomial, which allowed high-frequency noise to be suppressed. The second derivative of the polynomial was then calculated numerically. R-peaks were identified using a threshold detector applied to the differentiated signal. In the second stage, the quality of R-peak detection was checked by an expert using software that visualized the ECG and RR-intervals. During visual inspection, the expert manually corrected the positions of incorrectly detected R-peaks.

A sequence of RR-intervals (time intervals between successive R-peaks) was extracted from the ECG recordings. To obtain an equidistant time series, this sequence was approximated with cubic splines and resampled at 0.05 s intervals, resulting in an RR-interval signal with an effective sampling frequency of 20 Hz [38].

For each MFI block and for the BL and Post stages, HRV measures were calculated from the RR-interval signals according to the well-known recommendations [38]. The analyzed physiological measures included HR, RR-interval variance (D), the stress index (SI), power of the LF and HF components, total spectral power (TP), and the LF/HF ratio. LF, HF, and the LF/HF ratio were considered measures of the spectral characteristics of HRV and were not used for direct assessment of the activity of the sympathetic or parasympathetic branches of the autonomic nervous system.

To minimize inter-individual variability, all measures were normalized to their BL values according to the following formula:

(4) $$\Delta X_{i}=\frac{X_{i}-X_{BL}}{X_{BL}},$$

where $\Delta X_{i}$ is the normalized value of the metric in the *i*-th block, $X_{i}$ is its original value in the *i*-th block, and $X_{\mathrm{BL}}$ is its value in the BL stage.

This transformation made it possible to express HRV measures as proportions of their baseline level and to assess relative changes in physiological parameters. The normalized measures are denoted as $\Delta {\mathrm{HR}}_{i}$, $\Delta D_{i}$, $\Delta {\mathrm{SI}}_{i}$, $\Delta {\mathrm{LF}}_{i}$, $\Delta {\mathrm{HF}}_{i}$ and $\Delta \mathrm{LF}/{\mathrm{HF}}_{i}$, and their median values across the subjects are denoted as ΔHR, ΔD, ΔSI, ΔLF, ΔHF and ΔLF/HF, respectively.

### 4.3. Phases of Cognitive Performance

Based on the individual MFI dynamics, all MFI blocks were classified into three phases of cognitive performance: the adaptation phase (AP), the optimal performance phase (OP), and the fatigue phase (FP). This classification followed Hebb’s concept of activation and work curves and formal fatigue–recovery models describing the phases of cognitive performance [39,40].

The AP consisted of all initial blocks with ${\mathrm{IES}}_{i}<0$ up to the first block with ${\mathrm{IES}}_{i}\geq 0$. In other words, these were the initial blocks at the beginning of the recording in which the subject’s performance was below the individual mean, which corresponds to the classical notion of an initial AP of cognitive processes to the demands of the task [41]. Some subjects already showed above-average performance in the first block. Their performance decreased only as fatigue developed. For these subjects, the AP was considered absent.

The OP consisted of the blocks from the first block after AP or, if the AP was absent, from the first block of the recording up to the last block with ${\mathrm{IES}}_{i}\geq 0$. Thus, the OP was characterized by above-average cognitive performance with a stable balance between speed and accuracy, which is consistent with the established interpretation of effective cognitive performance [42,43].

The FP corresponded to a period of sustained performance decrease due to the development of mental fatigue [44]. It included all blocks from the end of the OP to the end of the recording. In several subjects, performance during the OP did not fall below the average level. In these cases, the FP was considered absent for those subjects.

It should be noted that this classification represents a relative division of individual cognitive performance dynamics because the IES was standardized relative to the complete recording of each subject. Therefore, the identified AP, OP, and FP characterize successive periods of changes in cognitive performance within a specific experimental session and are not considered independently validated physiological states.

### 4.4. Statistical Analysis

Statistical analysis was performed using linear mixed-effects models (LMMs) [45]. A separate LMM was fitted for each physiological measure (HR, D, SI, LF, HF, and LF/HF).

The fixed-effects part of each model included the categorical factors cognitive task (Task: SST/WMT), group according to the direction of the initial HR response (HR group: ΔHR ≥ 0/ΔHR < 0), and experimental phase (Phase), as well as their two-way interactions: Task × HR group, HR group × Phase, and Task × Phase. A random intercept for subject was included to account for the dependence of repeated observations within the same individual. For subjects who performed both cognitive tasks, the SST and WMT observations retained the same subject identifier, allowing their within-subject dependence to be accounted for by the random effect. This approach accommodated the partially overlapping sample design: 45 subjects performed the SST, 38 performed the WMT, and 35 performed both tasks, resulting in a total of 48 unique subjects.

Because the number of 15 min blocks comprising individual phases of cognitive performance varied between subjects, the original data were aggregated at the Subject × Task × Phase level before model fitting. For each subject, task, and phase, a single representative value was calculated as the median of the corresponding physiological measure across all 15 min blocks assigned to that phase. Thus, the unit of repeated observation in the LMM was the phase-level observation for the corresponding subject.

The presence or absence of the AP was determined exclusively from the IES dynamics. When the AP was identified, the median physiological value across all 15 min blocks assigned to the AP was used. When the AP was not identified, the first 15 min block of task performance was used as an operational physiological value representing the early task period for AP-related statistical comparisons. This procedure was used only for the analysis of physiological responses and did not alter the classification of the AP as present or absent based on cognitive performance. If a separate FP was absent, no substitute value was assigned, and the models were estimated using the available phase-level observations without imputation.

Model parameters were estimated using maximum likelihood. For each model-based comparison, an estimate (Estimate) characterizing the magnitude and direction of the difference between the compared conditions was calculated together with its 95% confidence interval (95% CI). In addition, a standardized effect size and its corresponding 95% CI were calculated. Two-sided *p*-values were obtained for all comparisons.

To control the family-wise error rate (FWER) associated with multiple comparisons, the *p*-values were adjusted using the Holm procedure [46]. Results were considered statistically significant at a Holm-adjusted *p* < 0.05. Quantitative data are reported as the median and interquartile range, Q2 (Q1; Q3).

Statistical analyses were performed in Python (version 3.12.5) using the SciPy (version 1.15.1), pandas (version 2.2.2), and statsmodels (version 0.15.0) libraries. The full results of the linear mixed-effects model analyses are presented in Supplementary Tables S1–S3. The sample sizes and distribution of subjects according to HR group and the presence or absence of cognitive performance phases are presented in Supplementary Figure S1.

Because the study sample had already been collected, a sensitivity power analysis was performed using G*Power 3.1.9.7 [47,48] rather than an a priori sample size calculation. The analysis was based on a two-tailed independent-samples *t*-test with α = 0.05 and statistical power of 0.80 and was used to determine the minimum detectable effect size given the actual group sizes.

## 5. Results

### 5.1. Validation of Fatigue Induction Protocols

**Figure 2:**
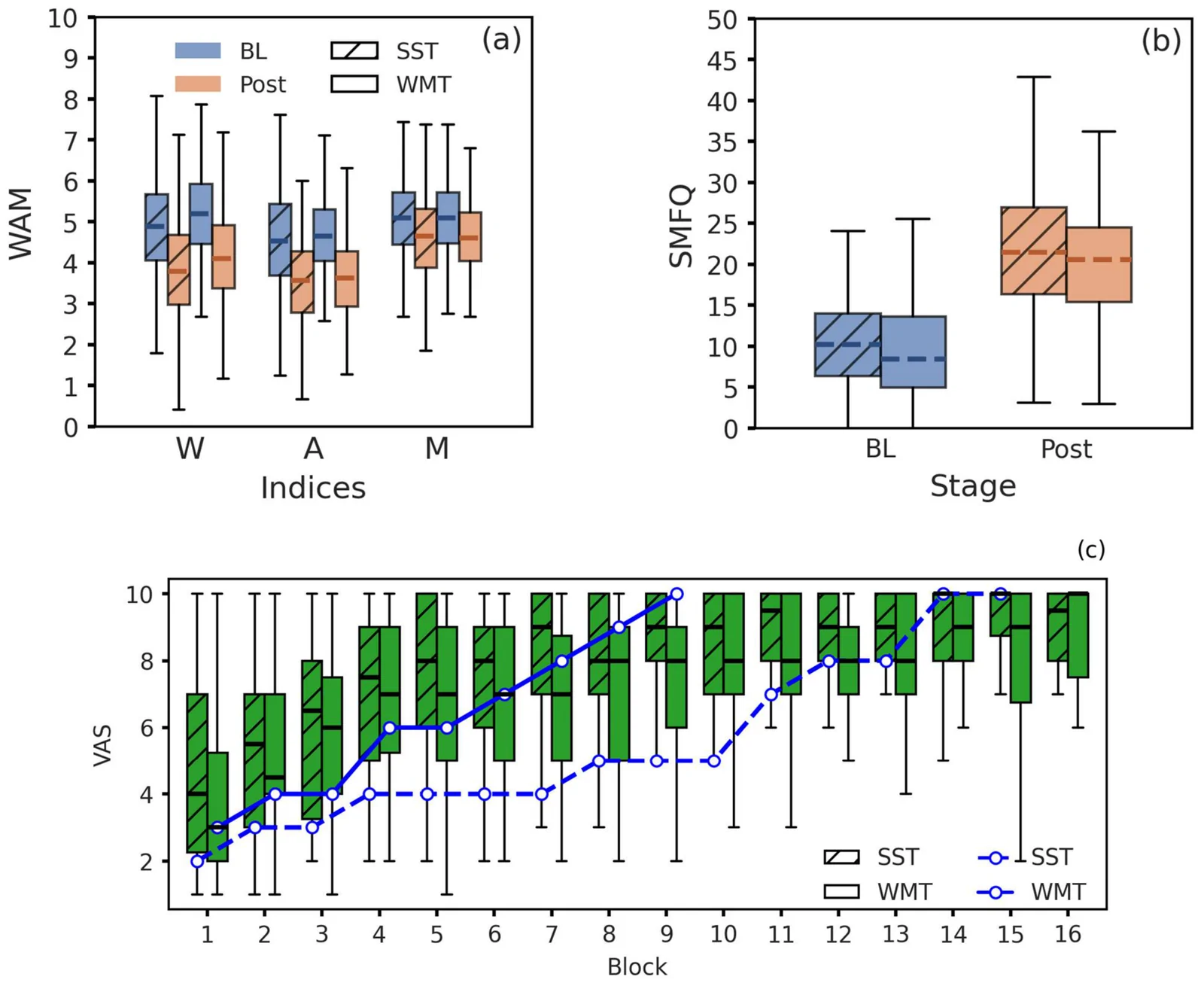
Dynamics of subjective fatigue ratings. (**a**) WAM scores before and after the SST and WMT. (**b**) SMFQ scores before and after the SST and WMT. (**c**) Dynamics of VAS fatigue ratings across successive 15 min blocks during the MFI stage in the SST and WMT. Hatching denotes SST, while the absence of hatching denotes WMT. The dashed and solid lines show the individual dynamics of subject #1 in the SST and WMT, respectively.

Figure 2 shows the dynamics of subjective fatigue ratings during the SST and the WMT, assessed using the WAM (Figure 2a), SMFQ (Figure 2b), and the VAS (Figure 2c). A statistically significant decrease in well-being, activity, and mood scores was observed after both tasks, with the median values decreasing by approximately the same value in both experiments (Figure 2a).

The subjective mental fatigue index derived from the SMFQ more than doubled after task performance (Figure 2b), indicating a shift from moderate to pronounced fatigue. The VAS dynamics showed a progressive increase in subjective fatigue level during task performance, reaching the maximum possible values (Figure 2c).

Comparison of the distributions of all subjective ratings between SST and WMT did not reveal statistically significant differences, indicating comparable induction of subjective fatigue by both types of tasks.

Thus, both cognitive tasks induced pronounced subjective fatigue, while its level was comparable during the SST and WMT.

### 5.2. Behavioral Performance Dynamics

**Figure 3:**
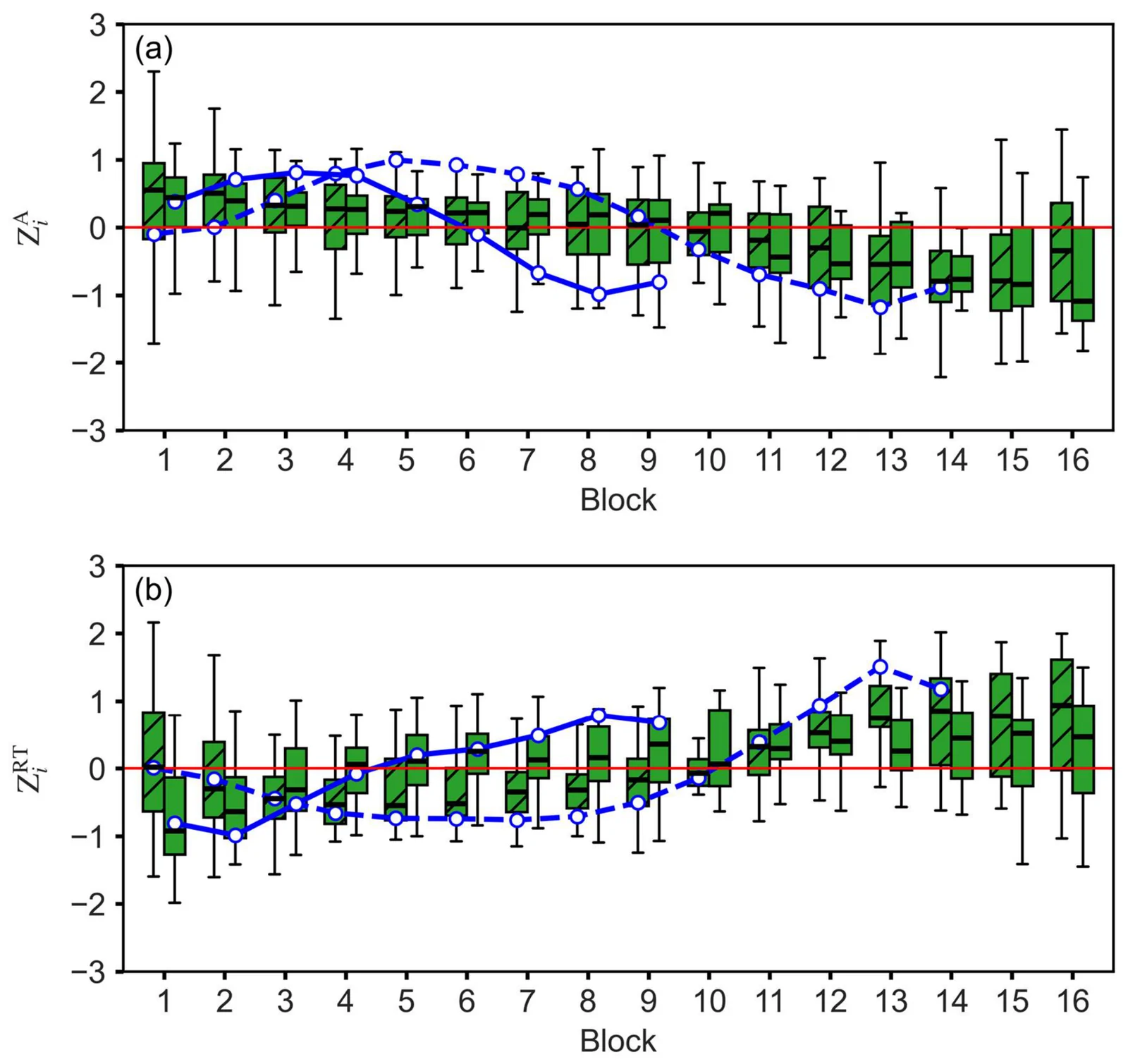

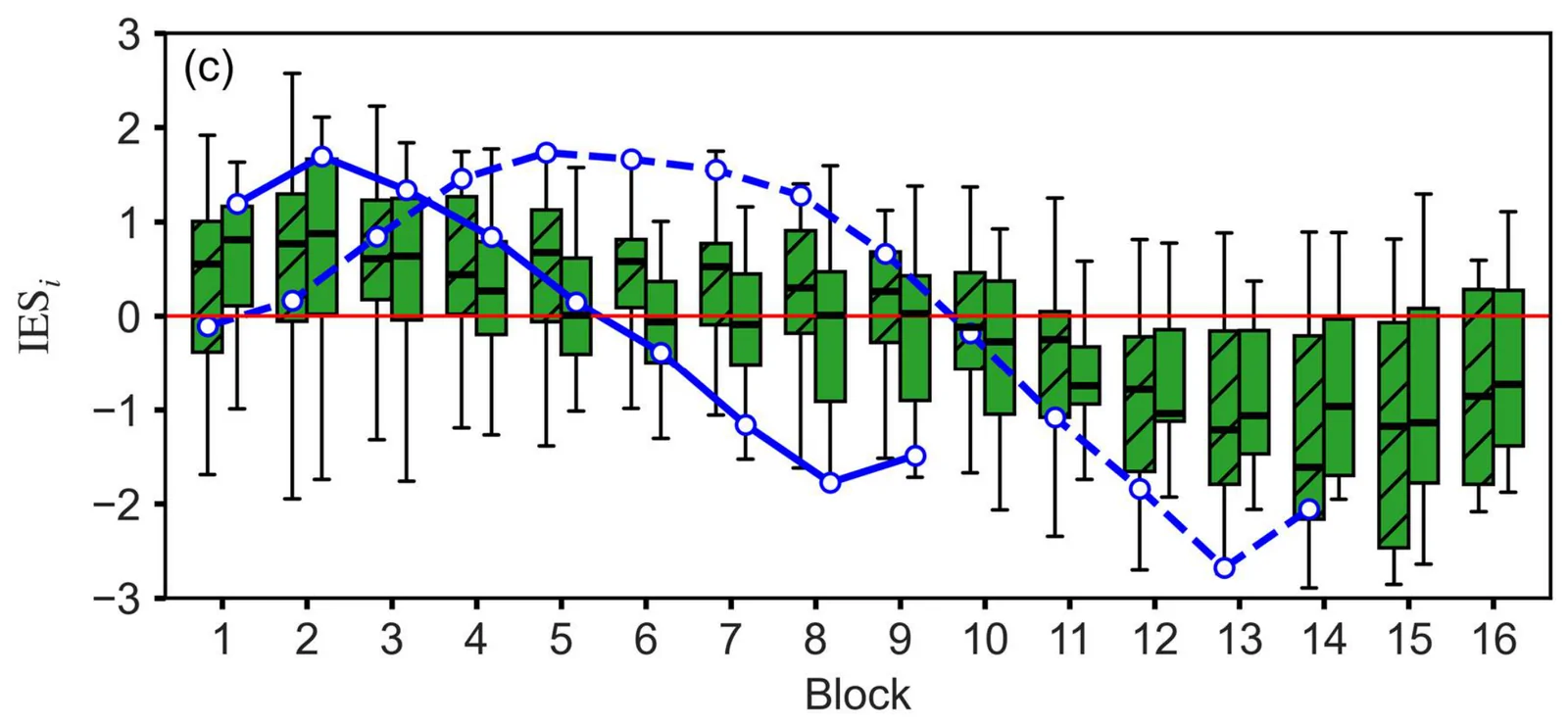
Dynamics of behavioral performance measures during prolonged mentally fatiguing tasks. (**a**) Distributions of standardized accuracy across blocks in SST and WMT. (**b**) Distributions of standardized reaction time across blocks in SST and WMT. (**c**) Distributions of the index of efficiency of mental performance (IES) across blocks in the SST and WMT. Dashed and solid blue lines show the individual dynamics of behavioral indices for subject #1 for SST and WMT, respectively. The red line serves as the threshold separating below-average from above-average performance.

Figure 3 presents boxplots showing the distributions of standardized accuracy (Figure 3a), standardized reaction time (Figure 3b), and the index of efficiency of mental performance (IES) (Figure 3c) in the SST and WMT. The individual dynamics is shown for subject #1.

On average, as the duration of the experiment increased, accuracy decreased (Figure 3a) and reaction time increased (Figure 3b), resulting in a decrease in the IES index (Figure 3c). No statistically significant differences in behavioral indices between the two types of tasks were found. The increase in the dispersion of averaged indices towards the end of the experiment can be explained by early task termination in some subjects, which reduced the ensemble size at longer duration of recordings.

Analysis of behavioral indices at the level of individual subjects (Figure 3) showed that in some cases a traditional pattern with clearly expressed AP, OP, and FP was observed. For subject #1, this pattern is present for both SST and WMT in Figure 3a and for the SST in Figure 3b,c. However, in a number of cases one or more phases were absent. For subject #1 (Figure 3), such a situation is observed for standardized reaction time and the index of IES. The AP is absent in these cases, further illustrating inter-individual differences in cognitive performance dynamics across the two tasks.

**Figure 4:**
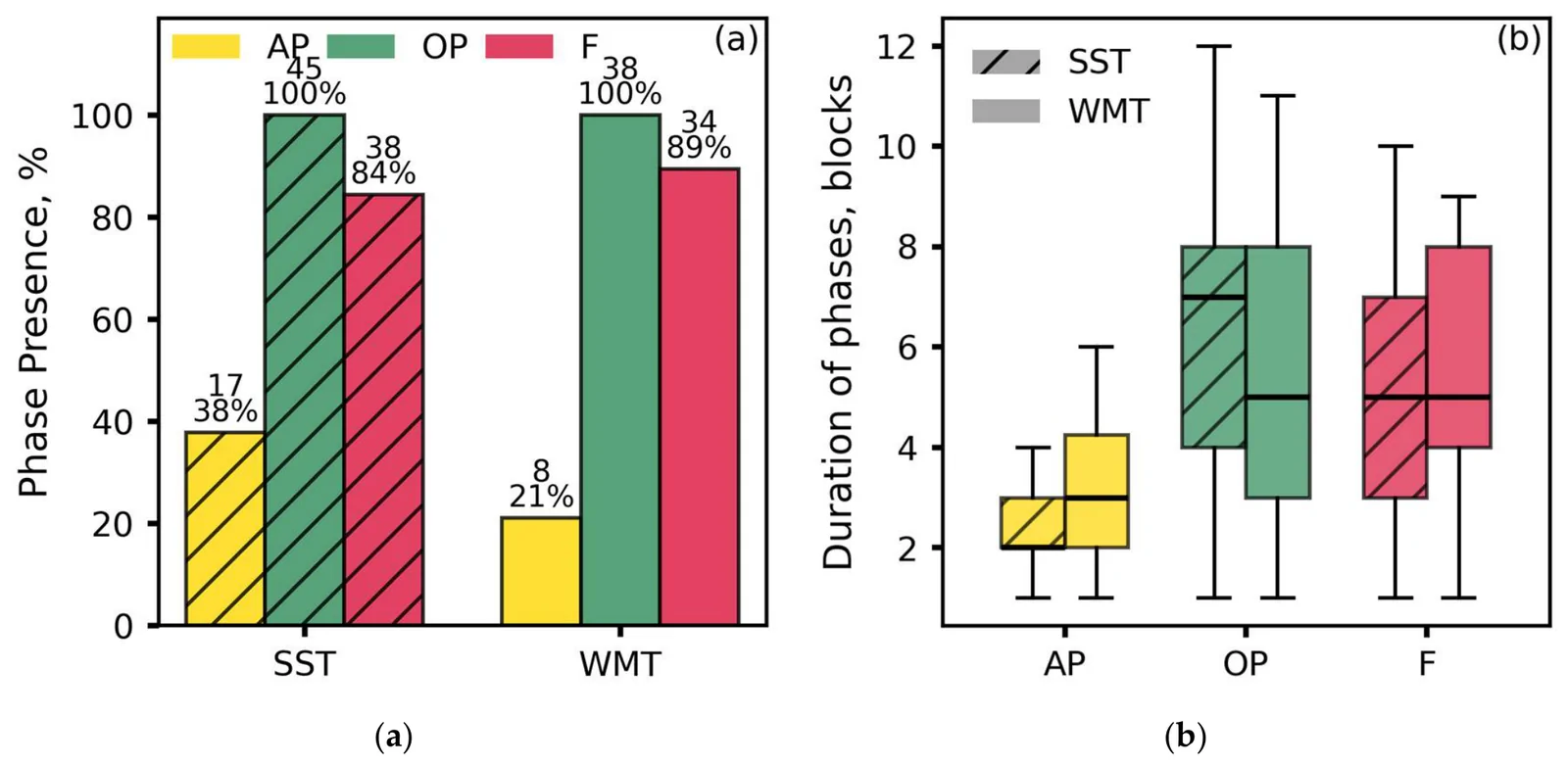
Statistics of the identified phases of cognitive performance in SST and WMT. (**a**) Proportion of subjects exhibiting each phase. (**b**) Durations of the phases. The boundaries of each boxplot correspond to the first and third quartiles, the line inside the box indicates the median, and the whiskers show the minimum and maximum values in the sample.

Figure 4a shows the proportion of subjects exhibiting the three phases of cognitive performance (AP, OP, and FP) in SST and WMT. The OP was present in 100% of subjects according to the classification procedure. The AP was identified in only 40% of subjects in SST and 21% in WMT, whereas the FP was observed in 84% of subjects in SST and 89% in WMT.

Figure 4b presents boxplots of the durations of the identified phases. The AP had the shortest duration, and its length was generally consistent with published estimates of adaptation time [49]. However, AP in subjects performing the WMT was, on average, significantly longer than in those performing the SST.

The duration of the OP varied across subjects, which may reflect individual differences in work capacity. At the group level, no differences in the duration of the OP were found between the two types of tasks, and no differences were observed for the FP, which was limited either by the maximum experiment duration or by the individual ability of subjects to continue the task.

Thus, cognitive performance decreased during both tasks. However, individual dynamics varied substantially: the OP was present in all subjects, whereas the AP and FP were not identified in all cases.

### 5.3. Analysis of Biosignals

The first key finding in the biosignal analysis was a decrease in HR in some subjects during the AP. This finding contrasts with most previous studies, which reported either an increase in HR or no significant change [50,51,52].

**Figure 5:**
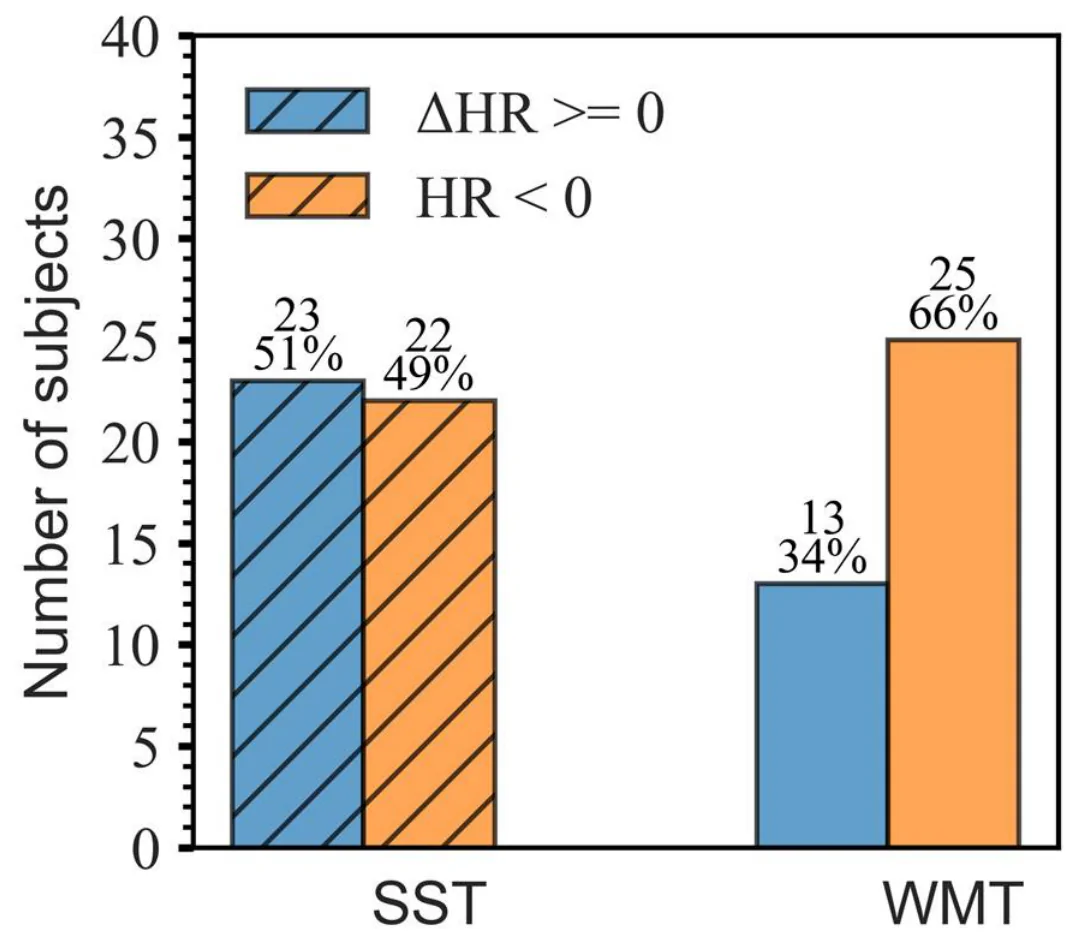
Distribution of subjects by pattern of HR dynamics during task performance. The numbers above the bars show the percentage of subjects with each pattern.

However, in our study a decrease in the median HR was the typical response (Figure 5). In the WMT, this pattern was observed in about half of the subjects, whereas in the SST it was seen in almost two-thirds of the sample.

**Figure 6:**
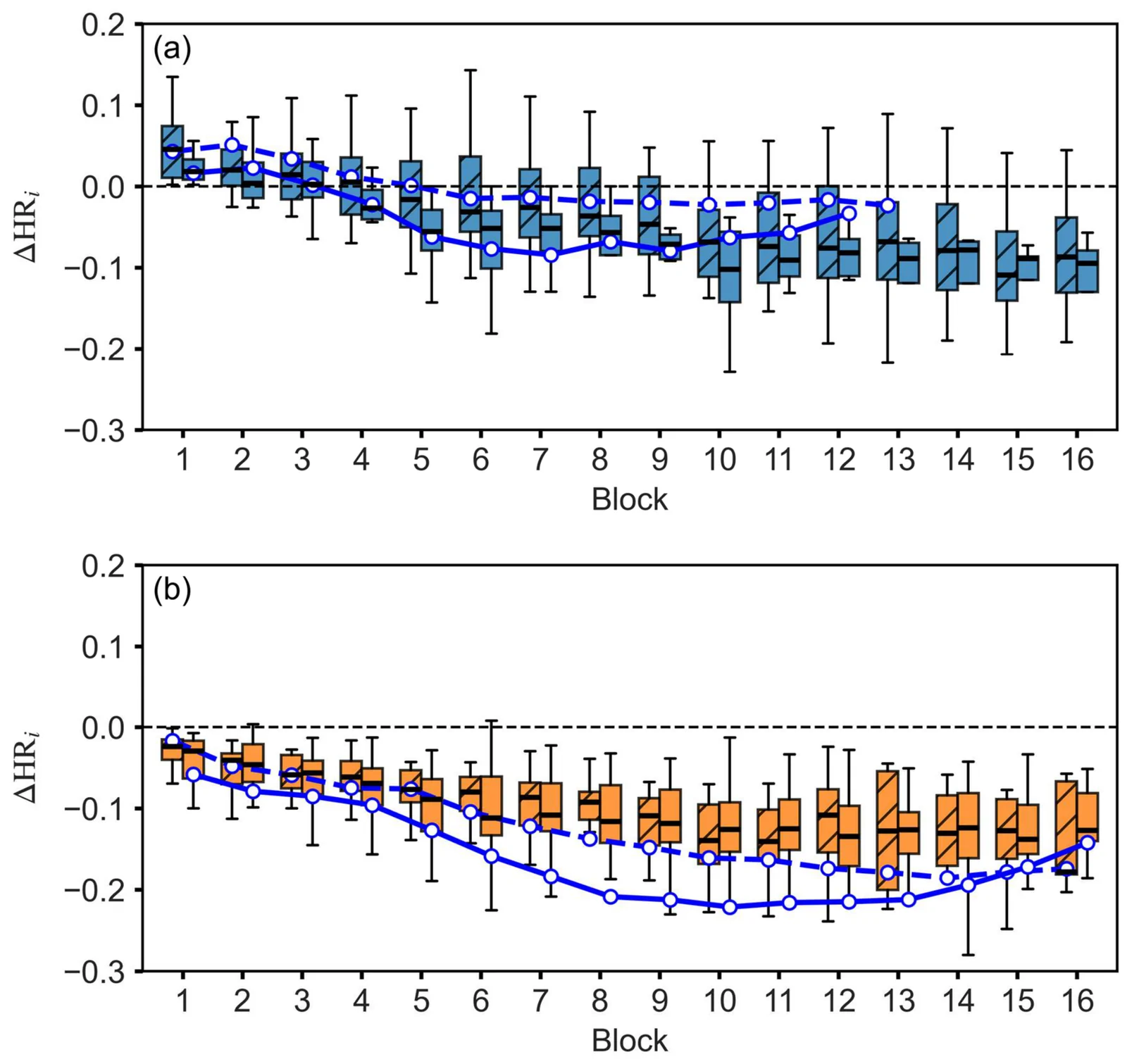
Distribution of subjects according to the direction of the initial HR response during the SST and WMT: (**a**) ΔHR ≥ 0 group; (**b**) ΔHR < 0 group. Hatching denotes SST, while the absence of hatching denotes WMT. The dashed and solid lines show the individual ΔHR dynamics of subjects #2 and #3 during the SST and WMT, respectively.

Figure 6 shows mean ΔHR across blocks for two subjects who completed both types of tasks. Subject #2 shows an initial increase in HR during the AP, followed by a decrease and subsequent stabilization. Subject #3 shows a monotonic decrease in HR during the AP and OP followed by a slight increase in HR in the FP. Analysis of mean ΔHR in the two groups with opposite initial ΔHR signs indicates that these patterns are typical.

Of the 35 subjects who performed both tasks, 18 showed the same HR response pattern in both tasks: 7 exhibited an increase in HR during the AP, and 11 showed a monotonic decrease. Among the remaining subjects, 11 showed ΔHR ≥ 0 in the AP during the SST and ΔHR < 0 in this phase during the WMT, whereas 6 subjects showed the opposite pattern (Supplementary Figure S1).

To test the hypothesis that the absence of an initial HR increase in some subjects (Figure 6) is linked to the absence of an AP, we calculated the statistics presented in Table 1 and Supplementary Figure S1. The table shows that although the proportion of subjects without an AP is slightly higher in the ΔHR < 0 group than in the ΔHR ≥ 0 group, this difference is small and may reflect sampling variability.

The obtained results allowed us to identify two patterns of the initial HR response to cognitive load: ΔHR ≥ 0 and ΔHR < 0. Therefore, in the subsequent analysis, the recordings were analyzed separately in the ΔHR ≥ 0 and ΔHR < 0 groups.

The sensitivity power analysis performed using G*Power [47,48] revealed that, with the obtained sample sizes, the minimum detectable standardized mean difference (Cohen’s d) was 0.86 for the SST comparison (n_1_ = 23, n_2_ = 22) and 0.98 for the WMT comparison (n_1_ = 13, n_2_ = 25). Thus, the study was adequately powered only to detect large effect sizes, whereas small-to-moderate effects may have remained undetected.

**Figure 7:**
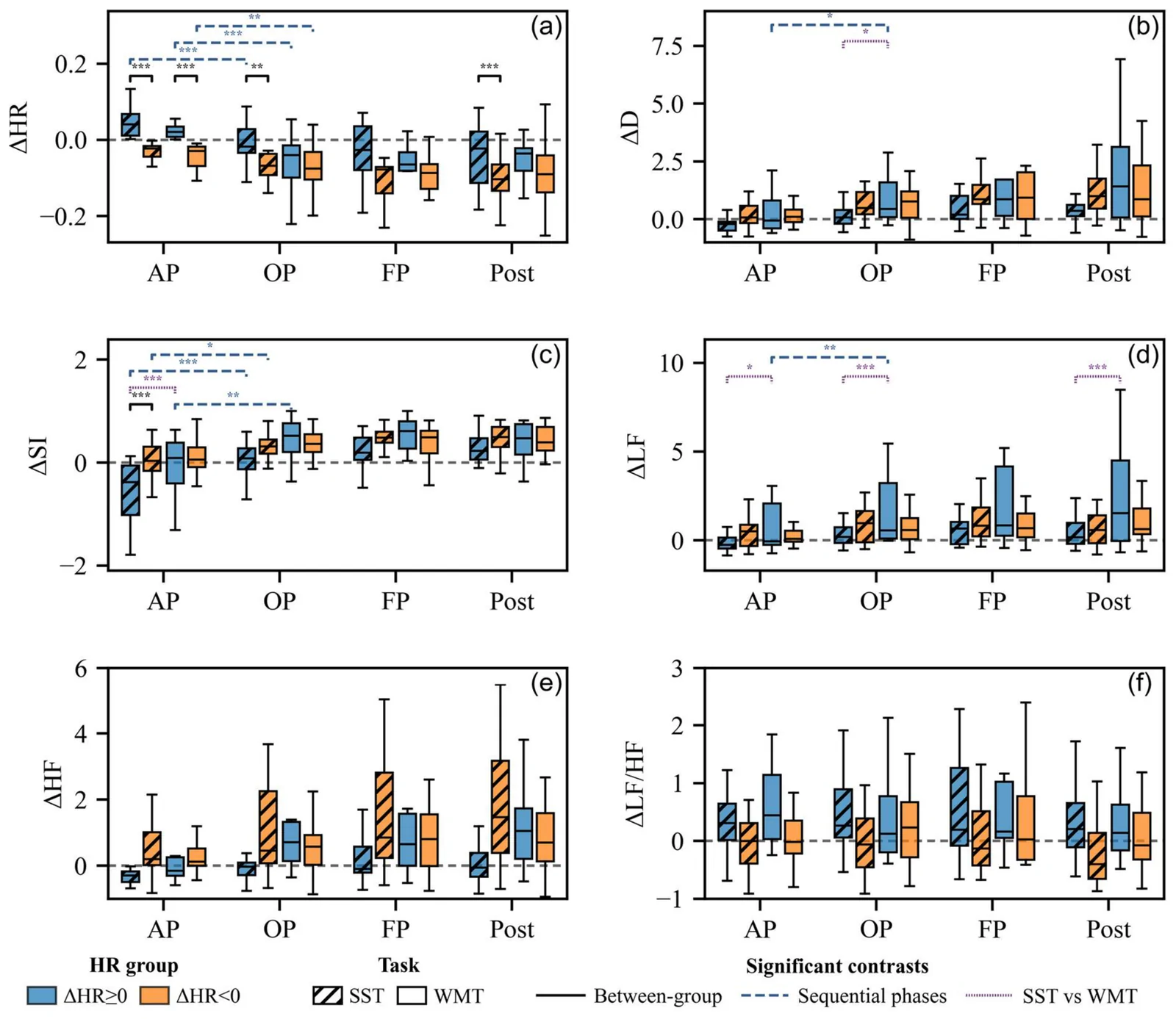
Dynamics of HRV measures normalized to the BL values across the AP, OP, FP, and Post stages in the ΔHR ≥ 0 and ΔHR < 0 groups: (**a**) ΔHR; (**b**) ΔD; (**c**) ΔSI; (**d**) ΔLF; (**e**) ΔHF; (**f**) ΔLF/HF. Colors indicate the direction of the initial HR response (ΔHR ≥ 0 or ΔHR < 0): when an individually identified AP was present, ΔHR was determined for this phase; when AP was absent, the first 15 min task block was used. Hatching denotes SST, while the absence of hatching denotes WMT. Brackets denote model-based comparisons. Asterisks indicate statistical significance after Holm correction: *p* < 0.05 (*), *p* < 0.01 (**), and *p* < 0.001 (***).

Figure 7 presents the dynamics of HRV measures normalized to the BL values during the AP, OP, FP, and Post stages. Detailed results of the statistical analysis are presented in Tables S1–S3 of the Supplementary Materials.

In the SST, the most pronounced pattern of between-group differences was observed at the early stage of task performance (Figure 7, Table S1). During the AP, the ΔHR ≥ 0 group was characterized by higher HR, lower SI and HF, and higher LF/HF compared with the ΔHR < 0 group. Differences in HR also persisted during the OP, FP, and Post stages. Thus, the direction of the initial HR response was associated with persistent between-group differences in HR throughout the subsequent phases of the SST and after task completion.

Analysis of successive between-phase changes (Figure 7, Table S2) in the SST showed that the main statistically significant changes were observed during the AP → OP transition. In the ΔHR ≥ 0 group, this transition was accompanied by a decrease in HR and an increase in SI. In the ΔHR < 0 group, a statistically significant increase in SI was observed during the AP → OP transition. No statistically significant changes were found for the subsequent OP → FP and FP → Post transitions.

In the WMT, between-group differences showed a different phase profile (Figure 7, Table S1). HR was higher in the ΔHR ≥ 0 group than in the ΔHR < 0 group during the AP and FP. LF values were higher in the ΔHR ≥ 0 group during the OP and FP, and LF/HF was higher during the AP, FP, and Post stages. No statistically significant between-group differences were found for D, SI, or HF.

Analysis of successive between-phase changes in the WMT also showed that statistically significant changes were associated with the AP → OP transition (Figure 7, Table S2). In both the ΔHR ≥ 0 and ΔHR < 0 groups, this transition was accompanied by a decrease in HR. In the ΔHR ≥ 0 group, it was additionally accompanied by an increase in SI. No statistically significant changes were found for the subsequent OP → FP and FP → Post transitions.

Comparison of physiological measures between the SST and WMT (Figure 7, Table S3) revealed between-task differences in the ΔHR ≥ 0 group. In this group, HR during the OP was higher in the SST than in the WMT. In contrast, D was lower in the SST during the OP, FP, and Post stages. During the AP, SI was also lower in the SST than in the WMT.

The most persistent between-task differences in the ΔHR ≥ 0 group were found for LF. LF values were lower in the SST than in the WMT during all analyzed phases: AP, OP, FP, and Post. For LF/HF, a statistically significant difference was observed during the Post stage, when its value was also lower in the SST than in the WMT. No statistically significant between-task differences were found for HF. In the ΔHR < 0 group, none of the differences between the SST and WMT remained statistically significant.

Thus, two patterns of the initial HR response (ΔHR ≥ 0 and ΔHR < 0) were identified, whereas the main changes in HRV measures were observed predominantly during the AP → OP transition. Differences between the SST and WMT indicate that physiological dynamics depend on the type of cognitive task performed.

## 6. Discussion

The present study investigated individual dynamics of cognitive performance and HRV indices during prolonged mental load. The use of two cognitive tasks and an experimental duration of up to 4 h (Figure 1) allowed us to analyze changes not only between the beginning and end of the load but also across individual phases of cognitive performance.

Analysis of subjective ratings confirmed the development of pronounced fatigue during both cognitive tasks (Figure 2). At the same time, the level of subjective fatigue was comparable between the SST and WMT, allowing differences in physiological dynamics to be considered in the context of individual response characteristics and the type of cognitive task performed.

The proposed integrated index of relative efficiency allowed us to analyze individual dynamics of cognitive performance and identify the AP, OP, and FP phases (Figure 3). In contrast to the classical concept of a sequential progression through adaptation, optimal performance, and fatigue phases [39,44], AP was identified in fewer than half of the participants, whereas OP was observed in all participants and FP in the majority of the participants (Figure 4). Among participants with an identified AP, its duration was, on average, longer during the WMT than during the SST (Figure 4b). These differences may be related to the characteristics of cognitive tasks with different structures. The longer AP during the WMT may reflect a longer period required to achieve stable performance in a working memory task that requires the maintenance and continuous updating of information [53]. The absence or short duration of AP may be associated with a greater individual ability to perform a particular type of task. However, individual abilities were not assessed in the present study. Overall, these findings primarily demonstrate pronounced interindividual variability in the transition to efficient cognitive performance and indicate that the AP → OP → FP sequence is not universal.

Another important finding was the identification of two patterns of the initial HR response to cognitive load: ΔHR ≥ 0 and ΔHR < 0 (Figure 5 and Figure 6). Analysis of the 35 participants who completed both tasks showed that the direction of ΔHR was the same in the SST and WMT in 18 participants, whereas it differed in 17 participants. This indicates that the direction of the initial HR response is not a stable individual characteristic but may depend on individual differences in cardiovascular and HRV dynamics as well as on the nature of the cognitive task performed.

The present findings are consistent with previous studies reporting different patterns of HR dynamics during prolonged cognitive load [18]. Most studies reported an increase in HR or no significant changes. However, some studies also observed a decrease in HR. Matuz et al. showed an initial increase in HR followed by a decrease in HR during prolonged performance of a cognitive task [19,54]. This was accompanied by an increase in vagally mediated HRV indices [54]. In another study, the decrease in HR did not correlate with indices of mental fatigue, leading the authors to suggest a possible contribution of other factors, including prolonged sitting [55]. Wittels et al. showed that high cognitive load was accompanied by an increase in HR, whereas low cognitive load was associated with a decrease in HR [56]. Thus, previous studies indicate that HR may either increase or decrease during prolonged cognitive load and that the direction of these changes depends on task conditions.

In this context, the identified ΔHR ≥ 0 and ΔHR < 0 patterns can be considered different variants of the physiological response to the onset of cognitive load. However, the specific mechanisms underlying these patterns were not assessed in the present study; therefore, their physiological interpretation requires further investigation.

When comparing our findings with previous studies [18,19,54,55,56], differences in analytical approaches should be considered. Most previous studies analyzed group-averaged measures, whereas individual HR changes in opposite directions may be partially obscured by such an approach. In the present study, accounting for the individual direction of the initial HR response allowed us to identify two patterns that might have remained undetected in analyses based solely on group-level data.

In addition to differences in HR, the HRV analysis showed that the most pronounced changes in the investigated physiological measures occurred during the early stage of prolonged cognitive load, predominantly in the AP-to-OP comparison (Figure 7). In subjects without an individually identified AP, the first 15 min task block served as the operational AP value for this physiological comparison. In contrast, the transition from OP to FP was not accompanied by statistically significant changes in the investigated HRV indices. Previous studies have demonstrated associations of HRV with cognitive control and the stability of cognitive task performance [57,58]. However, our findings indicate that the dynamics of the HRV measures do not directly follow the decline in cognitive performance and cannot be regarded as simple physiological markers of increasing mental fatigue. Differences between the SST and WMT further indicate that the physiological response depends on the nature of the task performed. Thus, assessment of mental fatigue using HR and HRV indices should take into account the individual initial response, the phase of cognitive performance, and the type of task performed.

The present findings indicate the value of further investigating personalized approaches to monitoring mental fatigue that account for changes in physiological indices relative to the individual baseline. Following appropriate validation, such approaches could potentially be implemented using wearable devices for monitoring functional state and adaptive management of cognitive load. The widespread use of devices that record heart rate, such as smartwatches, fitness trackers, and smart rings, suggests that, if reliable methods are developed, they could be implemented using existing devices. Alternatively, it is reasonable to expect that monitoring devices with the technical characteristics required for such analysis could be developed.

The strengths of the study include prolonged cognitive load lasting up to 4 h, the use of two different tasks, and the analysis of individual physiological dynamics. Identification of the AP, OP, and FP phases made it possible to assess changes in physiological indices not only between the beginning and end of the load but also across different phases of cognitive performance. The participation of a subset of volunteers in both experiments also made it possible to assess whether individual responses depended on the task performed.

Nevertheless, the study has several limitations. The participants were healthy young volunteers examined at a single center. This limits the generalizability of the findings to other age groups and clinical populations. In addition, the experimental tasks only partially reproduce the conditions of real-world prolonged mental activity. Despite the randomized order of the SST and WMT and the 4–5-week interval between experiments, the influence of repeated participation cannot be completely excluded.

Identification of the AP, OP, and FP phases was based on the IES standardized relative to the complete individual recording. Therefore, phase boundaries may have depended on the duration of the session and the time at which it was terminated. The identified phases should be regarded as relative periods of individual cognitive performance dynamics rather than independently validated physiological states of mental fatigue. Sensitivity analyses using alternative IES thresholds and independent criteria for phase identification were not performed and are needed for further validation of the approach.

Task duration varied between participants and was determined by the time at which the experiment was terminated. To reduce the influence of these differences, for each participant and each phase, the median physiological value across the corresponding 15 min blocks was calculated. This prevented participants with longer phases from receiving greater statistical weight. However, the influence of differences in experimental session duration cannot be completely excluded.

HR and HRV dynamics may also have been influenced by factors not directly related to cognitive load. These include prolonged sitting, physical inactivity, circadian changes, and sleepiness. Conducting the experiments in the morning reduced variability related to time of day. However, these factors were not directly assessed.

The effects of sex and baseline physiological state were not analyzed separately. Given the available sample size, further division of participants into subgroups will reduce the reliability of the statistical estimates. Investigation of these factors requires a larger sample.

## 7. Conclusions

The present study demonstrated pronounced interindividual variability in cognitive performance and in the dynamics of HRV measures during prolonged mental load. The sequence of the adaptation phase, optimal performance phase, and fatigue phase was not universal: the optimal performance phase was observed in all participants, whereas the adaptation phase and fatigue phase were not identified in all cases. The direction of the initial heart rate response also differed between participants and could vary within the same individual depending on the task performed.

The most pronounced changes in HRV indices were observed during the early stage of the load, predominantly during the transition from the adaptation phase to the optimal performance phase, whereas the transition from the optimal performance phase to the fatigue phase was not accompanied by significant changes in the investigated indices. Thus, the dynamics of HRV measures do not always correspond to the dynamics of cognitive performance and depend on both the individual response and the nature of the task performed. These findings highlight the need to account for individual physiological dynamics when studying prolonged mental load and may be useful for the further development of personalized approaches to monitoring mental fatigue.

## Figures and Tables

**Table 1:** Statistics for subjects without an AP based on the analysis of the index of efficiency of mental performance IES. Values are given as A/B (C), where A is the number of subjects without an AP, B is the total number of subjects in the group, and C is the proportion of subjects without an AP.

Group	Task	
	SST	WMT
ΔHR ≥ 0	12/23 (0.52)	10/13 (0.77)
ΔHR < 0	16/22 (0.73)	20/25 (0.80)

## Data Availability

The datasets generated during and/or analysed during the current study are not publicly available due to ethical restrictions but are available from the corresponding author on reasonable request.

## Supplementary Materials

The following supporting information can be downloaded at: https://www.mdpi.com/article/10.3390/life16091553/s1, Figure S1: Distribution of subjects according to phases of cognitive performance and patterns of the initial HR response; Supplementary Methods: Linear Mixed-Effects Models for the Analysis of Physiological Measures; Table S1: Between-Group Comparisons (ΔHR ≥ 0 and ΔHR < 0) Within Each Task and Phase; Table S2: Between-Phase Changes Within Each Task and ΔHR Group; Table S3: Between-Task Comparisons of the SST and WMT Within ΔHR Groups and Experimental Phases.
