# Supplementary material for: Individual Patterns of Heart Rate Variability During Prolonged Cognitive Load with Mental Fatigue Induction
Source: Life (Basel). 2026 Sep 16;16(9):1553. doi: 10.3390/life16091553 (PMC13608147; doi:10.3390/life16091553)
Supplement: Supplementary file 1 [file life-16-01553-s001.zip › life-4496991-supplementary.pdf]

Supplementary Materials

Individual Patterns of Heart Rate Variability during Prolonged Cognitive Load with Mental Fatigue Induction

Content of Complementary Materials

Supplementary Figure S1. Distribution of subjects according to phases of cognitive performance and patterns of the initial HR response
Supplementary Methods. Linear Mixed-Effects Models for the Analysis of Physiological Measures
Table S1. Between-Group Comparisons (ΔHR ≥ 0 and ΔHR < 0) Within Each Task and Phase
Table S2. Between-Phase Changes Within Each Task and ΔHR Group
Table S3. Between-Task Comparisons of the SST and WMT Within ΔHR Groups and Experimental Phases

Supplementary Figure S1. Distribution of subjects according to phases of cognitive performance and patterns of the initial HR response

Figure S1 summarizes the distribution of subjects according to the presence or absence of the AP and FP and the direction of the initial HR response (ΔHR ≥ 0 and ΔHR < 0) during the SST and WMT. The upper panel presents all subjects separately for the SST and WMT, whereas the lower panel includes only the 35 subjects who performed both cognitive tasks.

The Adaptation Phase (AP), Optimal Performance Phase (OP), and Fatigue Phase (FP) denote the phases of cognitive performance used in this study. ΔHR denotes the change in HR during the early period of task performance relative to the baseline level and was used to divide subjects into the ΔHR ≥ 0 and ΔHR < 0 groups. When an individually identified AP was present, ΔHR was determined for this phase; when the AP was absent, the first 15-minute block of task performance was used to determine the direction of the initial HR response. For subjects without an AP, the first block was used exclusively to determine ΔHR group membership and was not classified as the AP.

"AP present/absent" and "FP present/absent" denote the presence or absence of the corresponding individually identified phase. The proportions of subjects with and without the AP and FP within the ΔHR subgroups were calculated relative to the number of subjects in the corresponding ΔHR group. Because ΔHR group membership was determined separately for the SST and WMT, the same subject could belong to different ΔHR groups when performing the two cognitive tasks.

Summary Figure S1. A total of 48 healthy subjects participated in the study: 45 performed the SST protocol, 38 performed the WMT protocol, and 35 performed both tasks. In the SST experiment (n = 45), the AP, OP, and FP were identified in 17 (37.8%), 45 (100%), and 38 (84.4%) subjects, respectively. According to the direction of the initial HR response, 23 subjects (51.1%) were classified into the ΔHR ≥ 0 group and 22 (48.9%) into the ΔHR < 0 group. In the ΔHR ≥ 0 group, the AP was present in 11/23 (47.8%) and absent in 12/23 (52.2%) subjects, whereas the FP was present in 19/23 (82.6%) and absent in 4/23 (17.4%). In the ΔHR < 0 group, the AP was present in 6/22 (27.3%) and absent in 16/22 (72.7%) subjects; the FP was present in 19/22 (86.4%) and absent in 3/22 (13.6%).

In the WMT experiment (n = 38), the AP, OP, and FP were identified in 8 (21.1%), 38 (100%), and 34 (89.5%) subjects, respectively. Thirteen subjects (34.2%) showed ΔHR ≥ 0, whereas 25 (65.8%) showed ΔHR < 0. Among subjects with ΔHR ≥ 0, the AP was present in 3/13 (23.1%) and absent in 10/13 (76.9%), whereas the FP was present in all 13 subjects (100%). Among subjects with ΔHR < 0, the AP was present in 5/25 (20.0%) and absent in 20/25 (80.0%); the FP was present in 21/25 (84.0%) and absent in 4/25 (16.0%).

To assess the reproducibility of the HR response across different cognitive tasks, a separate analysis was performed for the 35 subjects who performed both the SST and WMT. During the SST, 18/35 (51.4%) subjects showed ΔHR ≥ 0 and 17/35 (48.6%) showed ΔHR < 0. During the WMT, the corresponding values were 12/35 (34.3%) and 23/35 (65.7%). The matrix comparing responses between the two tasks showed that 18/35 subjects (51.4%) had the same direction of the HR response in both experiments: 7 subjects showed ΔHR ≥ 0 during both tasks, whereas 11 showed ΔHR < 0 during both tasks. In contrast, in 17/35 subjects (48.6%), the direction of the HR response depended on the type of task: 11 subjects showed ΔHR ≥ 0 during the SST and ΔHR < 0 during the WMT, whereas 6 subjects showed the opposite pattern.

Thus, the OP was identified in all subjects in both experiments, whereas the presence of the AP showed considerably greater variability. The nearly equal proportions of subjects with the same and different directions of ΔHR during the SST and WMT indicate that, in a substantial proportion of subjects, the initial HR response was not a stable characteristic independent of the type of task performed. In addition, the presented distribution shows that the absence of the AP occurred in both ΔHR groups and, therefore, was not unambiguously associated with a decrease in HR.

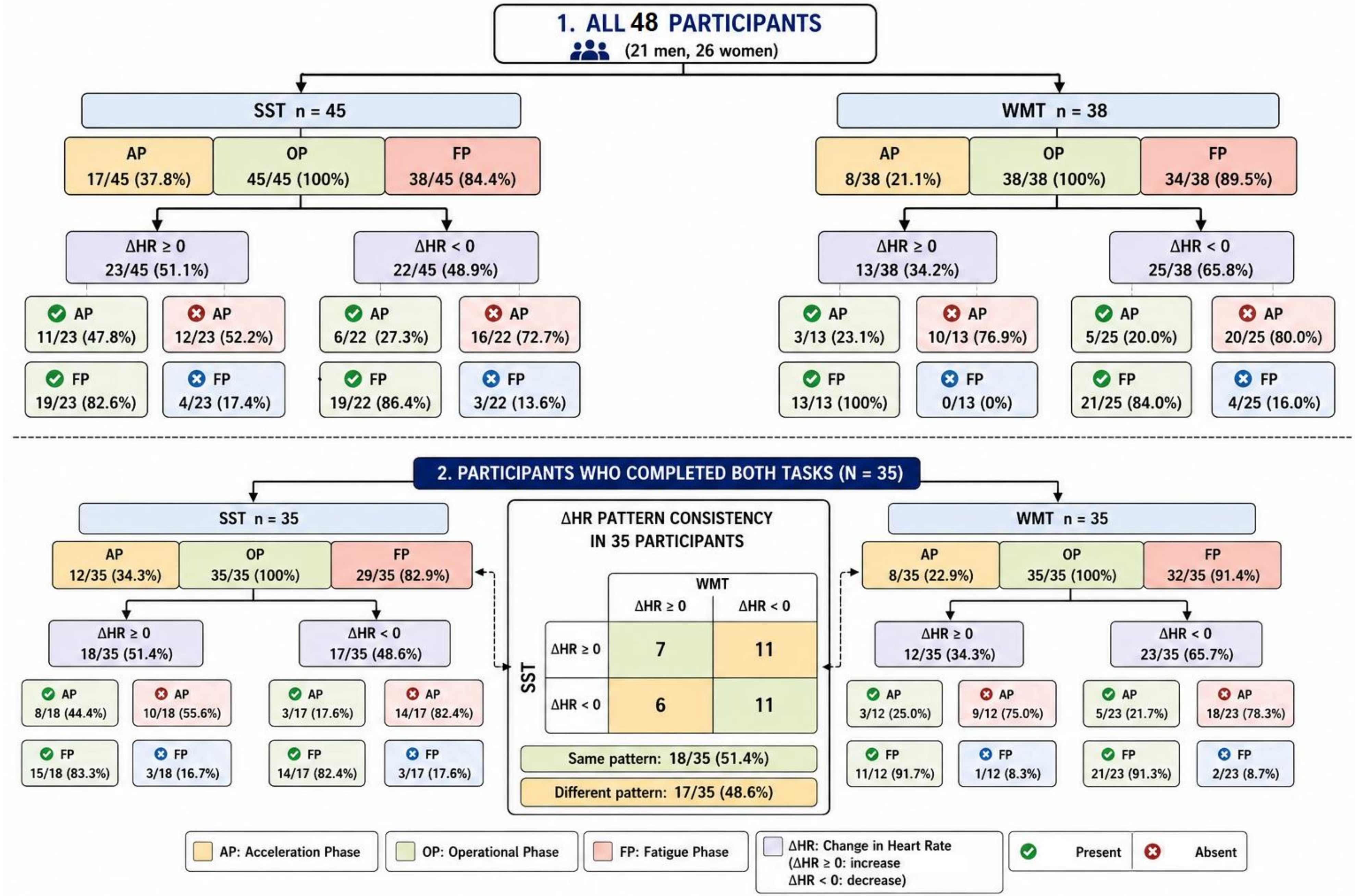

Figure S1. AP, OP, and FP denote the phases used in this study; ΔHR denotes the change in heart rate at the beginning of task performance. "AP yes/no" and "FP yes/no" denote the presence or absence of the corresponding phase. Percentages for AP and FP within the ΔHR subgroups were calculated relative to the total number of subjects in the corresponding ΔHR subgroup. The lower part of the figure presents only the 35 subjects who performed both tasks, the SST and WMT.

Supplementary Methods. Linear Mixed-Effects Models for the Analysis of Physiological Measures

Between-group, between-phase, and between-task differences in physiological measures were assessed using linear mixed-effects models (LMMs) [Bates et al., 2015].

Data preparation

For each subject, cognitive task, and phase, the median value of each physiological measure (HR, D, SI, LF, HF, and LF/HF) was calculated across the corresponding 15-min blocks. Thus, each Subject × Task × Phase combination was represented by a single aggregated value, irrespective of the number of blocks and the duration of the corresponding phase. This approach prevented subjects with longer phases from receiving greater statistical weight solely because they contributed a larger number of 15-min blocks.

If the adaptation phase (AP) was identified based on the individual IES dynamics, the median value of the corresponding physiological measure across all 15-min blocks assigned to AP was used for the physiological analysis. If AP was absent, the physiological value from the first 15-min block of task performance was used for AP-related comparisons. This operational definition allowed the early physiological response to task onset to be characterized in all subjects.

Importantly, the use of the first task block in subjects without AP was restricted exclusively to the analysis of physiological measures and did not alter the classification of cognitive performance phases based on IES. Subjects in whom AP was not identified from the IES dynamics remained classified as AP absent in all analyses describing the occurrence and duration of cognitive performance phases. Thus, a missing AP was not imputed at the level of cognitive performance classification; in these subjects, only the physiological value used for AP-related comparisons was operationally defined using the first task block.

If the fatigue phase (FP) was absent, the corresponding physiological value was neither generated nor imputed; the subject's remaining available observations were retained in the analysis.

Structure of the LMMs

A separate LMM was fitted for each physiological measure. The fixed effects included Task (SST/WMT), HR group ($\Delta HR \geq 0/\Delta HR < 0$), Phase, and the two-way interactions Task × HR group, HR group × Phase, and Task × Phase.

The model structure can be expressed as: Y ~ Task + HR group + Phase + Task × HR group + HR group × Phase + Task × Phase + (1 | Subject), where Y denotes the physiological measure being analyzed.

To account for repeated observations within subjects, a random intercept for Subject was included, with Subject used as the grouping factor. For subjects who performed both cognitive tasks, the SST and WMT observations retained the same Subject identifier, thereby accounting for within-subject dependence and the partially overlapping sample design.

HR group was determined separately for the SST and WMT according to the direction of the initial HR response. Consequently, the same subject could belong to different HR groups in the two tasks.

Model parameters were estimated using maximum likelihood (ML). Missing phase-level observations were not imputed; the models were fitted using the available observations.

Model contrasts

After fitting the LMMs, three prespecified families of linear contrasts of the fixed effects were evaluated:

- between-group contrasts (Table S1): $\Delta HR \geq 0 - \Delta HR < 0$;
- successive between-phase contrasts (Table S2): OP − AP, FP − OP, and Post − FP;
- between-task contrasts (Table S3): SST − WMT.

A positive Estimate indicated a higher model-estimated value of the physiological measure in the first condition of the contrast, whereas a negative Estimate indicated a higher value in the second condition. Estimate represented the model-based estimate of the corresponding contrast derived from the fitted LMM and therefore could differ from the simple difference between the observed medians.

Supplementary Tables S1–S3 also report descriptive values as Median (Q1; Q3). For between-phase contrasts, n paired denotes the number of subjects with available observations in both phases being compared. For between-task contrasts, n overlap denotes the number of the same subjects with corresponding observations available in both the SST and WMT.

Effect size and correction for multiple comparisons

For each contrast, the model-based Estimate, its 95% confidence interval (95% CI), and a two-sided *p*-value were calculated.

The standardized effect size (Std. effect) was calculated as:

Std. effect = Estimate / residual SD

where residual SD is the residual standard deviation of the corresponding LMM. The term residual standard deviation follows the terminology of Bates et al. A 95% CI was also calculated for the standardized effect size.

To control the family-wise error rate within each prespecified family of contrasts for each physiological measure, the Holm procedure was applied [Holm, 1979]. The correction was performed separately for each physiological measure within each prespecified family of contrasts presented in Supplementary Tables S1–S3. Both unadjusted and Holm-adjusted p-values are reported. Differences were considered statistically significant at Holm-adjusted $p < 0.05$.

Table S1. Between-Group Comparisons ($\Delta HR \geq 0$ and $\Delta HR < 0$) Within Each Task and Phase

Table S1 presents the results of between-group comparisons of physiological measures between subjects differing in the direction of the initial heart rate response ($\Delta HR \geq 0$ and $\Delta HR < 0$), separately for each cognitive task (SST and WMT) and each experimental phase (BL, AP, OP, FP, and Post). The analysis was performed using linear mixed-effects models (LMMs), accounting for repeated measurements from the same subject and inter-individual variability. For each physiological measure, differences between the $\Delta HR \geq 0$ and $\Delta HR < 0$ groups were assessed within the same task and experimental phase. Each row of the table addresses a specific question: does the selected physiological measure differ between the $\Delta HR \geq 0$ and $\Delta HR < 0$ groups during a given phase of a specific cognitive task?

Summary of Table S1. After Holm correction, the most consistent between-group differences were observed for HR. In the SST, HR differed between the $\Delta HR \geq 0$ and $\Delta HR < 0$ groups during the AP, OP, FP, and Post stages, whereas in the WMT, statistically significant differences were found during the AP and FP. Significant between-group differences in HRV measures were more specific to individual phases and tasks: SI and HF differed between the groups during the AP in the SST; LF differed during the OP and FP in the WMT; and LF/HF differed during the AP in the SST and during the AP, FP, and Post stages in the WMT. After correction for multiple comparisons, no statistically significant between-group differences were found for D.

Table S1. Between-Group Comparisons ($\Delta HR \geq 0$ and $\Delta HR < 0$) Within Each Task and Phase

Metric	Task	Phase	n $\Delta HR \geq 0$	$\Delta HR \geq 0$ Median (Q1; Q3)	n $\Delta HR < 0$	$\Delta HR < 0$ Median (Q1; Q3)	Estimate [95% CI]	Std. effect [95% CI]	*p*-value	Holm-adjusted *p*
HR	SST	AP	23	0.041 (0.012; 0.068)	22	-0.022 (-0.044; -0.015)	0.080 [0.055; 0.106]	1.673 [1.141; 2.206]	<0.001	**<0.001**
HR	SST	OP	23	-0.017 (-0.034; 0.028)	22	-0.067 (-0.092; -0.036)	0.049 [0.023; 0.075]	1.013 [0.476; 1.550]	<0.001	**0.001**
HR	SST	FP	19	-0.027 (-0.079; 0.036)	19	-0.078 (-0.140; -0.071)	0.065 [0.038; 0.092]	1.352 [0.790; 1.915]	<0.001	**<0.001**
HR	SST	Post	23	-0.023 (-0.113; 0.022)	22	-0.103 (-0.133; -0.064)	0.054 [0.028; 0.080]	1.124 [0.592; 1.657]	<0.001	**<0.001**
HR	WMT	AP	13	0.021 (0.008; 0.036)	25	-0.029 (-0.069; -0.018)	0.060 [0.034; 0.087]	1.258 [0.699; 1.817]	<0.001	**<0.001**
HR	WMT	OP	12	-0.040 (-0.099; -0.014)	25	-0.075 (-0.103; -0.031)	0.029 [0.002; 0.056]	0.598 [0.032; 1.163]	0.038	0.115
HR	WMT	FP	13	-0.064 (-0.081; -0.032)	21	-0.086 (-0.129; -0.063)	0.045 [0.017; 0.073]	0.937 [0.363; 1.510]	0.001	**0.007**
HR	WMT	Post	13	-0.035 (-0.080; -0.021)	25	-0.090 (-0.137; -0.040)	0.034 [0.007; 0.061]	0.709 [0.151; 1.268]	0.013	0.051
D	SST	AP	23	-0.199 (-0.485; -0.109)	22	0.087 (-0.159; 0.588)	-0.791 [-1.398; -0.184]	-0.678 [-1.198; -0.158]	0.011	0.107
D	SST	OP	23	0.091 (-0.184; 0.412)	22	0.493 (0.224; 1.179)	-0.327 [-0.939; 0.286]	-0.280 [-0.805; 0.245]	0.295	1.000
D	SST	FP	19	0.208 (0.016; 1.016)	19	0.877 (0.665; 1.499)	-0.235 [-0.878; 0.407]	-0.202 [-0.752; 0.349]	0.473	1.000
D	SST	Post	23	0.373 (0.137; 0.632)	22	1.013 (0.463; 1.760)	-0.343 [-0.950; 0.264]	-0.294 [-0.814; 0.226]	0.268	1.000
D	WMT	AP	13	-0.045 (-0.389; 0.824)	25	0.115 (-0.116; 0.423)	0.163 [-0.476; 0.803]	0.140 [-0.408; 0.688]	0.617	1.000
D	WMT	OP	12	0.448 (0.106; 1.609)	25	0.785 (0.067; 1.216)	0.628 [-0.020; 1.275]	0.538 [-0.017; 1.093]	0.058	0.429
D	WMT	FP	13	0.878 (0.157; 1.721)	21	0.941 (0.018; 2.043)	0.719 [0.061; 1.377]	0.616 [0.052; 1.180]	0.032	0.290
D	WMT	Post	13	1.432 (0.089; 3.137)	25	0.866 (0.133; 2.331)	0.611 [-0.028; 1.251]	0.524 [-0.024; 1.072]	0.061	0.429
SI	SST	AP	23	-0.376 (-1.017; -0.055)	22	0.037 (-0.158; 0.309)	-0.476 [-0.697; -0.254]	-1.091 [-1.599; -0.584]	<0.001	**<0.001**
SI	SST	OP	23	0.086 (-0.130; 0.277)	22	0.313 (0.174; 0.451)	-0.083 [-0.306; 0.140]	-0.190 [-0.701; 0.322]	0.468	1.000
SI	SST	FP	19	0.195 (0.053; 0.484)	19	0.485 (0.387; 0.599)	-0.166 [-0.401; 0.068]	-0.381 [-0.919; 0.156]	0.165	0.992
SI	SST	Post	23	0.229 (0.063; 0.472)	22	0.499 (0.303; 0.691)	-0.156 [-0.377; 0.065]	-0.358 [-0.865; 0.150]	0.167	0.992
SI	WMT	AP	13	0.089 (-0.403; 0.390)	25	0.058 (-0.087; 0.299)	-0.208 [-0.441; 0.025]	-0.476 [-1.010; 0.058]	0.081	0.726
SI	WMT	OP	12	0.526 (0.209; 0.768)	25	0.365 (0.208; 0.550)	0.186 [-0.050; 0.422]	0.425 [-0.116; 0.967]	0.123	0.987
SI	WMT	FP	13	0.617 (0.274; 0.803)	21	0.491 (0.183; 0.623)	0.102 [-0.139; 0.342]	0.234 [-0.318; 0.785]	0.406	1.000
SI	WMT	Post	13	0.475 (0.155; 0.745)	25	0.396 (0.234; 0.696)	0.112 [-0.121; 0.345]	0.257 [-0.277; 0.792]	0.345	1.000
LF	SST	AP	23	-0.242 (-0.450; 0.164)	22	0.520 (-0.306; 0.886)	-0.654 [-1.321; 0.012]	-0.509 [-1.028; 0.010]	0.054	0.326
LF	SST	OP	23	0.194 (-0.138; 0.736)	22	0.977 (-0.099; 1.668)	-0.225 [-0.897; 0.447]	-0.175 [-0.698; 0.348]	0.512	1.000
LF	SST	FP	19	0.670 (-0.203; 1.043)	19	0.838 (0.228; 1.851)	-0.177 [-0.882; 0.529]	-0.138 [-0.687; 0.411]	0.623	1.000
LF	SST	Post	23	0.179 (-0.196; 0.987)	22	0.580 (-0.154; 1.409)	-0.333 [-1.000; 0.334]	-0.259 [-0.778; 0.260]	0.327	0.982
LF	WMT	AP	13	-0.051 (-0.250; 2.077)	25	0.088 (-0.073; 0.542)	0.600 [-0.103; 1.302]	0.467 [-0.080; 1.013]	0.094	0.471
LF	WMT	OP	12	0.573 (0.110; 3.239)	25	0.587 (0.072; 1.249)	1.029 [0.318; 1.740]	0.801 [0.248; 1.354]	0.005	**0.041**
LF	WMT	FP	13	0.843 (0.271; 4.168)	21	0.696 (0.180; 1.518)	1.077 [0.354; 1.801]	0.838 [0.276; 1.401]	0.004	**0.035**
LF	WMT	Post	13	1.537 (-0.032; 4.501)	25	0.642 (0.356; 1.800)	0.921 [0.219; 1.624]	0.717 [0.170; 1.263]	0.010	0.081
HF	SST	AP	23	-0.295 (-0.487; -0.170)	22	0.203 (0.022; 1.016)	-2.610 [-4.188; -1.032]	-0.849 [-1.362; -0.336]	0.001	**0.012**
HF	SST	OP	23	-0.022 (-0.279; 0.096)	22	0.454 (0.081; 2.256)	-1.736 [-3.327; -0.146]	-0.565 [-1.082; -0.047]	0.032	0.292
HF	SST	FP	19	-0.093 (-0.208; 0.578)	19	0.852 (0.238; 2.815)	-1.081 [-2.752; 0.590]	-0.352 [-0.895; 0.192]	0.205	1.000
HF	SST	Post	23	-0.059 (-0.331; 0.395)	22	1.474 (0.387; 3.182)	-1.673 [-3.251; -0.096]	-0.544 [-1.057; -0.031]	0.038	0.301
HF	WMT	AP	13	-0.147 (-0.301; 0.255)	25	0.121 (-0.000; 0.520)	-1.487 [-3.148; 0.174]	-0.484 [-1.024; 0.057]	0.079	0.556
HF	WMT	OP	12	0.712 (0.143; 1.332)	25	0.577 (0.030; 0.930)	-0.613 [-2.296; 1.069]	-0.199 [-0.747; 0.348]	0.475	1.000
HF	WMT	FP	13	0.661 (0.000; 1.581)	21	0.811 (-0.004; 1.557)	0.042 [-1.671; 1.755]	0.014 [-0.543; 0.571]	0.962	1.000
HF	WMT	Post	13	1.052 (0.209; 1.736)	25	0.706 (0.133; 1.592)	-0.550 [-2.212; 1.111]	-0.179 [-0.719; 0.361]	0.516	1.000
LF/HF	SST	AP	23	0.307 (0.018; 0.646)	22	0.003 (-0.391; 0.311)	0.495 [0.156; 0.833]	0.777 [0.246; 1.309]	0.004	**0.033**
LF/HF	SST	OP	23	0.266 (0.063; 0.893)	22	-0.058 (-0.454; 0.389)	0.295 [-0.047; 0.636]	0.463 [-0.074; 1.000]	0.091	0.272
LF/HF	SST	FP	19	0.196 (-0.082; 1.263)	19	-0.131 (-0.422; 0.518)	0.457 [0.100; 0.815]	0.719 [0.157; 1.281]	0.012	0.073

**Metric**	**Task**	**Phase**	**n ΔHR≥0**	**ΔHR≥0 Median (Q1; Q3)**	**n ΔHR<0**	**ΔHR<0 Median (Q1; Q3)**	**Estimate [95% CI]**	**Std. effect [95% CI]**	***p*-value**	**Holm-adjusted *p***
LF/HF	SST	Post	23	0.209 (-0.107; 0.656)	22	-0.398 (-0.656; 0.139)	0.367 [0.029; 0.705]	0.577 [0.045; 1.108]	0.034	0.134
LF/HF	WMT	AP	13	0.448 (0.035; 1.145)	25	-0.014 (-0.218; 0.351)	0.626 [0.270; 0.981]	0.983 [0.424; 1.542]	<0.001	**0.006**
LF/HF	WMT	OP	12	0.125 (-0.194; 0.773)	25	0.232 (-0.282; 0.670)	0.426 [0.065; 0.786]	0.669 [0.103; 1.235]	0.021	0.103
LF/HF	WMT	FP	13	0.166 (0.053; 1.023)	21	0.027 (-0.326; 0.775)	0.588 [0.223; 0.954]	0.924 [0.350; 1.499]	0.002	**0.015**
LF/HF	WMT	Post	13	0.142 (-0.164; 0.632)	25	-0.073 (-0.321; 0.491)	0.498 [0.142; 0.854]	0.782 [0.223; 1.341]	0.006	**0.043**

Table S2. Between-Phase Changes Within Each Task and ΔHR Group

Table S2 presents the results of successive comparisons of the phases of cognitive performance within each task (SST and WMT) and separately within the two groups defined according to the direction of the initial heart rate response (ΔHR ≥ 0 and ΔHR < 0). The analysis was performed using linear mixed-effects models (LMMs). For each physiological measure, changes between successive phases were assessed: AP→OP, OP→FP, and FP→Post. Each row of the table addresses a specific question: does the selected physiological measure change during the transition from one phase to the next within a given group of subjects performing a specific cognitive task?

Summary of Table S2. After Holm correction, all six statistically significant successive between-phase changes were identified during the AP→OP transition. Three of these corresponded to a decrease in HR—in SST/ΔHR ≥ 0, WMT/ΔHR ≥ 0, and WMT/ΔHR < 0—and the other three corresponded to an increase in SI in SST/ΔHR ≥ 0, SST/ΔHR < 0, and WMT/ΔHR ≥ 0. No statistically significant successive changes in the investigated measures were found for the OP→FP and FP→Post transitions after Holm correction.

Table S2. Between-phase contrasts within each task and ΔHR group

Metric	Task	HR group	Contrast	n earlier	Earlier Median (Q1; Q3)	n later	Later Median (Q1; Q3)	n paired	Estimate [95% CI]	Std. effect [95% CI]	*p*-value	Holm-adjusted *p*
HR	SST	ΔHR≥0	AP → OP	23	0.041 (0.012; 0.068)	23	-0.017 (-0.034; 0.028)	23	-0.064 [-0.089; -0.039]	-1.328 [-1.842; -0.813]	<0.001	**<0.001**
HR	SST	ΔHR≥0	OP → FP	23	-0.017 (-0.034; 0.028)	19	-0.027 (-0.079; 0.036)	19	-0.019 [-0.045; 0.007]	-0.403 [-0.943; 0.136]	0.143	0.856
HR	SST	ΔHR≥0	FP → Post	19	-0.027 (-0.079; 0.036)	23	-0.023 (-0.113; 0.022)	19	-0.005 [-0.031; 0.020]	-0.114 [-0.652; 0.425]	0.679	1.000
HR	SST	ΔHR<0	AP → OP	22	-0.022 (-0.044; -0.015)	22	-0.067 (-0.092; -0.036)	22	-0.032 [-0.057; -0.007]	-0.667 [-1.190; -0.145]	0.012	0.099
HR	SST	ΔHR<0	OP → FP	22	-0.067 (-0.092; -0.036)	19	-0.078 (-0.140; -0.071)	19	-0.036 [-0.062; -0.010]	-0.743 [-1.287; -0.199]	0.007	0.067
HR	SST	ΔHR<0	FP → Post	19	-0.078 (-0.140; -0.071)	22	-0.103 (-0.133; -0.064)	19	0.005 [-0.021; 0.032]	0.114 [-0.428; 0.657]	0.680	1.000
HR	WMT	ΔHR≥0	AP → OP	13	0.021 (0.008; 0.036)	12	-0.040 (-0.099; -0.014)	12	-0.072 [-0.102; -0.043]	-1.502 [-2.119; -0.885]	<0.001	**<0.001**
HR	WMT	ΔHR≥0	OP → FP	12	-0.040 (-0.099; -0.014)	13	-0.064 (-0.081; -0.032)	12	-0.005 [-0.035; 0.025]	-0.107 [-0.734; 0.521]	0.739	1.000
HR	WMT	ΔHR≥0	FP → Post	13	-0.064 (-0.081; -0.032)	13	-0.035 (-0.080; -0.021)	13	-0.004 [-0.034; 0.026]	-0.079 [-0.698; 0.540]	0.803	1.000
HR	WMT	ΔHR<0	AP → OP	25	-0.029 (-0.069; -0.018)	25	-0.075 (-0.103; -0.031)	25	-0.040 [-0.064; -0.016]	-0.842 [-1.340; -0.343]	<0.001	**0.009**
HR	WMT	ΔHR<0	OP → FP	25	-0.075 (-0.103; -0.031)	21	-0.086 (-0.129; -0.063)	21	-0.021 [-0.047; 0.004]	-0.446 [-0.968; 0.076]	0.094	0.657
HR	WMT	ΔHR<0	FP → Post	21	-0.086 (-0.129; -0.063)	25	-0.090 (-0.137; -0.040)	21	0.007 [-0.018; 0.032]	0.149 [-0.372; 0.670]	0.575	1.000
D	SST	ΔHR≥0	AP → OP	23	-0.199 (-0.485; -0.109)	23	0.091 (-0.184; 0.412)	23	0.567 [-0.033; 1.168]	0.486 [-0.028; 1.001]	0.064	0.704
D	SST	ΔHR≥0	OP → FP	23	0.091 (-0.184; 0.412)	19	0.208 (0.016; 1.016)	19	0.461 [-0.168; 1.090]	0.395 [-0.144; 0.934]	0.151	1.000
D	SST	ΔHR≥0	FP → Post	19	0.208 (0.016; 1.016)	23	0.373 (0.137; 0.632)	19	0.178 [-0.450; 0.806]	0.153 [-0.385; 0.690]	0.578	1.000
D	SST	ΔHR<0	AP → OP	22	0.087 (-0.159; 0.588)	22	0.493 (0.224; 1.179)	22	0.103 [-0.507; 0.713]	0.088 [-0.435; 0.611]	0.741	1.000
D	SST	ΔHR<0	OP → FP	22	0.493 (0.224; 1.179)	19	0.877 (0.665; 1.499)	19	0.370 [-0.265; 1.004]	0.317 [-0.227; 0.860]	0.253	1.000
D	SST	ΔHR<0	FP → Post	19	0.877 (0.665; 1.499)	22	1.013 (0.463; 1.760)	19	0.286 [-0.347; 0.919]	0.245 [-0.297; 0.787]	0.376	1.000
D	WMT	ΔHR≥0	AP → OP	13	-0.045 (-0.389; 0.824)	12	0.448 (0.106; 1.609)	12	1.002 [0.283; 1.722]	0.859 [0.242; 1.476]	0.006	0.076
D	WMT	ΔHR≥0	OP → FP	12	0.448 (0.106; 1.609)	13	0.878 (0.157; 1.721)	12	0.285 [-0.446; 1.017]	0.245 [-0.383; 0.872]	0.445	1.000
D	WMT	ΔHR≥0	FP → Post	13	0.878 (0.157; 1.721)	13	1.432 (0.089; 3.137)	13	0.076 [-0.646; 0.798]	0.065 [-0.553; 0.684]	0.836	1.000
D	WMT	ΔHR<0	AP → OP	25	0.115 (-0.116; 0.423)	25	0.785 (0.067; 1.216)	25	0.538 [-0.044; 1.120]	0.461 [-0.038; 0.960]	0.070	0.704
D	WMT	ΔHR<0	OP → FP	25	0.785 (0.067; 1.216)	21	0.941 (0.018; 2.043)	21	0.194 [-0.415; 0.802]	0.166 [-0.355; 0.687]	0.532	1.000
D	WMT	ΔHR<0	FP → Post	21	0.941 (0.018; 2.043)	25	0.866 (0.133; 2.331)	21	0.184 [-0.423; 0.791]	0.158 [-0.363; 0.678]	0.552	1.000
SI	SST	ΔHR≥0	AP → OP	23	-0.376 (-1.017; -0.055)	23	0.086 (-0.130; 0.277)	23	0.750 [0.525; 0.974]	1.719 [1.205; 2.234]	<0.001	**<0.001**
SI	SST	ΔHR≥0	OP → FP	23	0.086 (-0.130; 0.277)	19	0.195 (0.053; 0.484)	19	0.113 [-0.122; 0.348]	0.259 [-0.280; 0.798]	0.346	1.000
SI	SST	ΔHR≥0	FP → Post	19	0.195 (0.053; 0.484)	23	0.229 (0.063; 0.472)	19	-0.023 [-0.257; 0.212]	-0.052 [-0.590; 0.486]	0.850	1.000
SI	SST	ΔHR<0	AP → OP	22	0.037 (-0.158; 0.309)	22	0.313 (0.174; 0.451)	22	0.357 [0.129; 0.585]	0.818 [0.295; 1.341]	0.002	**0.022**
SI	SST	ΔHR<0	OP → FP	22	0.313 (0.174; 0.451)	19	0.485 (0.387; 0.599)	19	0.196 [-0.040; 0.433]	0.450 [-0.093; 0.993]	0.104	0.832
SI	SST	ΔHR<0	FP → Post	19	0.485 (0.387; 0.599)	22	0.499 (0.303; 0.691)	19	-0.033 [-0.269; 0.203]	-0.075 [-0.617; 0.466]	0.785	1.000
SI	WMT	ΔHR≥0	AP → OP	13	0.089 (-0.403; 0.390)	12	0.526 (0.209; 0.768)	12	0.447 [0.178; 0.716]	1.024 [0.408; 1.641]	0.001	**0.012**
SI	WMT	ΔHR≥0	OP → FP	12	0.526 (0.209; 0.768)	13	0.617 (0.274; 0.803)	12	0.140 [-0.134; 0.413]	0.321 [-0.306; 0.947]	0.316	1.000
SI	WMT	ΔHR≥0	FP → Post	13	0.617 (0.274; 0.803)	13	0.475 (0.155; 0.745)	13	-0.044 [-0.313; 0.226]	-0.100 [-0.718; 0.519]	0.752	1.000
SI	WMT	ΔHR<0	AP → OP	25	0.058 (-0.087; 0.299)	25	0.365 (0.208; 0.550)	25	0.053 [-0.164; 0.271]	0.123 [-0.376; 0.621]	0.630	1.000
SI	WMT	ΔHR<0	OP → FP	25	0.365 (0.208; 0.550)	21	0.491 (0.183; 0.623)	21	0.223 [-0.004; 0.451]	0.512 [-0.009; 1.033]	0.054	0.486
SI	WMT	ΔHR<0	FP → Post	21	0.491 (0.183; 0.623)	25	0.396 (0.234; 0.696)	21	-0.054 [-0.281; 0.173]	-0.123 [-0.643; 0.397]	0.642	1.000
LF	SST	ΔHR≥0	AP → OP	23	-0.242 (-0.450; 0.164)	23	0.194 (-0.138; 0.736)	23	0.627 [-0.035; 1.288]	0.487 [-0.027; 1.002]	0.063	0.633
LF	SST	ΔHR≥0	OP → FP	23	0.194 (-0.138; 0.736)	19	0.670 (-0.203; 1.043)	19	0.288 [-0.405; 0.981]	0.224 [-0.315; 0.763]	0.415	1.000
LF	SST	ΔHR≥0	FP → Post	19	0.670 (-0.203; 1.043)	23	0.179 (-0.196; 0.987)	19	-0.288 [-0.979; 0.404]	-0.224 [-0.762; 0.314]	0.415	1.000
LF	SST	ΔHR<0	AP → OP	22	0.520 (-0.306; 0.886)	22	0.977 (-0.099; 1.668)	22	0.197 [-0.475; 0.869]	0.153 [-0.369; 0.676]	0.565	1.000
LF	SST	ΔHR<0	OP → FP	22	0.977 (-0.099; 1.668)	19	0.838 (0.228; 1.851)	19	0.240 [-0.459; 0.938]	0.187 [-0.357; 0.730]	0.501	1.000
LF	SST	ΔHR<0	FP → Post	19	0.838 (0.228; 1.851)	22	0.580 (-0.154; 1.409)	19	-0.131 [-0.828; 0.565]	-0.102 [-0.645; 0.440]	0.712	1.000
LF	WMT	ΔHR≥0	AP → OP	13	-0.051 (-0.250; 2.077)	12	0.573 (0.110; 3.239)	12	1.121 [0.329; 1.914]	0.872 [0.256; 1.489]	0.006	0.067
LF	WMT	ΔHR≥0	OP → FP	12	0.573 (0.110; 3.239)	13	0.843 (0.271; 4.168)	12	0.109 [-0.697; 0.914]	0.084 [-0.543; 0.712]	0.792	1.000
LF	WMT	ΔHR≥0	FP → Post	13	0.843 (0.271; 4.168)	13	1.537 (-0.032; 4.501)	13	0.128 [-0.667; 0.924]	0.100 [-0.519; 0.719]	0.752	1.000
LF	WMT	ΔHR<0	AP → OP	25	0.088 (-0.073; 0.542)	25	0.587 (0.072; 1.249)	25	0.692 [0.051; 1.333]	0.538 [0.040; 1.037]	0.034	0.377
LF	WMT	ΔHR<0	OP → FP	25	0.587 (0.072; 1.249)	21	0.696 (0.180; 1.518)	21	0.060 [-0.610; 0.730]	0.047 [-0.474; 0.568]	0.860	1.000
LF	WMT	ΔHR<0	FP → Post	21	0.696 (0.180; 1.518)	25	0.642 (0.356; 1.800)	21	0.285 [-0.384; 0.953]	0.222 [-0.299; 0.742]	0.404	1.000
HF	SST	ΔHR≥0	AP → OP	23	-0.295 (-0.487; -0.170)	23	-0.022 (-0.279; 0.096)	23	0.353 [-1.229; 1.935]	0.115 [-0.400; 0.629]	0.662	1.000
HF	SST	ΔHR≥0	OP → FP	23	-0.022 (-0.279; 0.096)	19	-0.093 (-0.208; 0.578)	19	0.310 [-1.347; 1.967]	0.101 [-0.438; 0.640]	0.714	1.000

Metric	Task	HR group	Contrast	n earlier	Earlier Median (Q1; Q3)	n later	Later Median (Q1; Q3)	n paired	Estimate [95% CI]	Std. effect [95% CI]	*p*-value	Holm-adjusted *p*
HF	SST	ΔHR≥0	FP → Post	19	-0.093 (-0.208; 0.578)	23	-0.059 (-0.331; 0.395)	19	0.166 [-1.488; 1.820]	0.054 [-0.484; 0.592]	0.844	1.000
HF	SST	ΔHR<0	AP → OP	22	0.203 (0.022; 1.016)	22	0.454 (0.081; 2.256)	22	-0.520 [-2.128; 1.087]	-0.169 [-0.692; 0.354]	0.526	1.000
HF	SST	ΔHR<0	OP → FP	22	0.454 (0.081; 2.256)	19	0.852 (0.238; 2.815)	19	-0.345 [-2.015; 1.325]	-0.112 [-0.655; 0.431]	0.685	1.000
HF	SST	ΔHR<0	FP → Post	19	0.852 (0.238; 2.815)	22	1.474 (0.387; 3.182)	19	0.758 [-0.908; 2.425]	0.247 [-0.295; 0.789]	0.373	1.000
HF	WMT	ΔHR≥0	AP → OP	13	-0.147 (-0.301; 0.255)	12	0.712 (0.143; 1.332)	12	1.362 [-0.534; 3.258]	0.443 [-0.174; 1.060]	0.159	1.000
HF	WMT	ΔHR≥0	OP → FP	12	0.712 (0.143; 1.332)	13	0.661 (0.000; 1.581)	12	-0.168 [-2.096; 1.760]	-0.055 [-0.682; 0.572]	0.865	1.000
HF	WMT	ΔHR≥0	FP → Post	13	0.661 (0.000; 1.581)	13	1.052 (0.209; 1.736)	13	-0.335 [-2.237; 1.568]	-0.109 [-0.728; 0.510]	0.730	1.000
HF	WMT	ΔHR<0	AP → OP	25	0.121 (-0.000; 0.520)	25	0.577 (0.030; 0.930)	25	0.488 [-1.045; 2.021]	0.159 [-0.340; 0.657]	0.533	1.000
HF	WMT	ΔHR<0	OP → FP	25	0.577 (0.030; 0.930)	21	0.811 (-0.004; 1.557)	21	-0.823 [-2.426; 0.779]	-0.268 [-0.789; 0.254]	0.314	1.000
HF	WMT	ΔHR<0	FP → Post	21	0.811 (-0.004; 1.557)	25	0.706 (0.133; 1.592)	21	0.258 [-1.342; 1.857]	0.084 [-0.436; 0.604]	0.752	1.000
LF/HF	SST	ΔHR≥0	AP → OP	23	0.307 (0.018; 0.646)	23	0.266 (0.063; 0.893)	23	-0.014 [-0.341; 0.314]	-0.022 [-0.536; 0.493]	0.934	1.000
LF/HF	SST	ΔHR≥0	OP → FP	23	0.266 (0.063; 0.893)	19	0.196 (-0.082; 1.263)	19	0.134 [-0.209; 0.477]	0.211 [-0.329; 0.750]	0.444	1.000
LF/HF	SST	ΔHR≥0	FP → Post	19	0.196 (-0.082; 1.263)	23	0.209 (-0.107; 0.656)	19	-0.263 [-0.606; 0.079]	-0.414 [-0.952; 0.124]	0.132	1.000
LF/HF	SST	ΔHR<0	AP → OP	22	0.003 (-0.391; 0.311)	22	-0.058 (-0.454; 0.389)	22	0.186 [-0.147; 0.519]	0.293 [-0.230; 0.816]	0.273	1.000
LF/HF	SST	ΔHR<0	OP → FP	22	-0.058 (-0.454; 0.389)	19	-0.131 (-0.422; 0.518)	19	-0.029 [-0.375; 0.317]	-0.045 [-0.589; 0.499]	0.871	1.000
LF/HF	SST	ΔHR<0	FP → Post	19	-0.131 (-0.422; 0.518)	22	-0.398 (-0.656; 0.139)	19	-0.173 [-0.518; 0.172]	-0.272 [-0.815; 0.271]	0.326	1.000
LF/HF	WMT	ΔHR≥0	AP → OP	13	0.448 (0.035; 1.145)	12	0.125 (-0.194; 0.773)	12	-0.092 [-0.484; 0.301]	-0.144 [-0.761; 0.473]	0.647	1.000
LF/HF	WMT	ΔHR≥0	OP → FP	12	0.125 (-0.194; 0.773)	13	0.166 (0.053; 1.023)	12	0.132 [-0.267; 0.531]	0.207 [-0.420; 0.835]	0.517	1.000
LF/HF	WMT	ΔHR≥0	FP → Post	13	0.166 (0.053; 1.023)	13	0.142 (-0.164; 0.632)	13	0.024 [-0.370; 0.418]	0.038 [-0.581; 0.657]	0.905	1.000
LF/HF	WMT	ΔHR<0	AP → OP	25	-0.014 (-0.218; 0.351)	25	0.232 (-0.282; 0.670)	25	0.108 [-0.209; 0.426]	0.170 [-0.328; 0.669]	0.503	1.000
LF/HF	WMT	ΔHR<0	OP → FP	25	0.232 (-0.282; 0.670)	21	0.027 (-0.326; 0.775)	21	-0.031 [-0.363; 0.301]	-0.048 [-0.570; 0.473]	0.856	1.000
LF/HF	WMT	ΔHR<0	FP → Post	21	0.027 (-0.326; 0.775)	25	-0.073 (-0.321; 0.491)	21	0.114 [-0.217; 0.446]	0.180 [-0.341; 0.700]	0.499	1.000

Table S3. Between-Task Comparisons of the SST and WMT Within ΔHR Groups and Experimental Phases

Table S3 presents the results of comparisons of physiological measures between the two cognitive tasks—the Serial Subtraction Task (SST) and Working Memory Task (WMT)—separately for the groups defined according to the direction of the initial heart rate response (ΔHR ≥ 0 and ΔHR < 0) and for each experimental phase. Between-task differences were assessed using linear mixed-effects models (LMMs), accounting for repeated measurements from the same subject and the partially overlapping SST and WMT samples. Each row of Table S3 shows whether the corresponding physiological measure differs between the SST and WMT within a specific ΔHR group and experimental phase, accounting for the repeated-measures structure and partial overlap of the samples.

Summary of Table S3. After Holm correction, 10 between-task comparisons remained statistically significant, all in the ΔHR ≥ 0 group: one for HR, three for D, one for SI, four for LF, and one for LF/HF. No statistically significant between-task differences were found for HF. In the ΔHR < 0 group, none of the physiological measures differed significantly between the SST and WMT after correction for multiple comparisons. Thus, between-task differences in HR and HRV measures were observed predominantly in subjects in the ΔHR ≥ 0 group.

Table S3. Task contrasts (SST vs WMT) within ΔHR group and phase

Metric	HR group	Phase	n SST	SST Median (Q1; Q3)	n WMT	WMT Median (Q1; Q3)	n overlap	Estimate [95% CI]	Std. effect [95% CI]	*p*-value	Holm-adjusted p
HR	ΔHR≥0	BL	23	0.000 (0.000; 0.000)	13	0.000 (0.000; 0.000)	7	0.013 [-0.013; 0.039]	0.275 [-0.262; 0.812]	0.316	1.000
HR	ΔHR≥0	AP	23	0.041 (0.012; 0.068)	13	0.021 (0.008; 0.036)	7	0.033 [0.007; 0.058]	0.678 [0.141; 1.215]	0.013	0.121
HR	ΔHR≥0	OP	23	-0.017 (-0.034; 0.028)	12	-0.040 (-0.099; -0.014)	7	0.041 [0.015; 0.067]	0.852 [0.309; 1.395]	0.002	**0.021**
HR	ΔHR≥0	FP	19	-0.027 (-0.079; 0.036)	13	-0.064 (-0.081; -0.032)	5	0.027 [-0.000; 0.054]	0.555 [-0.003; 1.113]	0.051	0.410
HR	ΔHR≥0	Post	23	-0.023 (-0.113; 0.022)	13	-0.035 (-0.080; -0.021)	7	0.025 [-0.001; 0.051]	0.521 [-0.017; 1.058]	0.058	0.410
HR	ΔHR<0	BL	22	0.000 (0.000; 0.000)	25	0.000 (0.000; 0.000)	10	-0.007 [-0.031; 0.017]	-0.141 [-0.643; 0.362]	0.583	1.000
HR	ΔHR<0	AP	22	-0.022 (-0.044; -0.015)	25	-0.029 (-0.069; -0.018)	10	0.013 [-0.012; 0.037]	0.262 [-0.240; 0.765]	0.306	1.000
HR	ΔHR<0	OP	22	-0.067 (-0.092; -0.036)	25	-0.075 (-0.103; -0.031)	10	0.021 [-0.003; 0.045]	0.437 [-0.069; 0.943]	0.091	0.544
HR	ΔHR<0	FP	19	-0.078 (-0.140; -0.071)	21	-0.086 (-0.129; -0.063)	8	0.007 [-0.019; 0.032]	0.140 [-0.391; 0.670]	0.605	1.000
HR	ΔHR<0	Post	22	-0.103 (-0.133; -0.064)	25	-0.090 (-0.137; -0.040)	10	0.005 [-0.019; 0.029]	0.105 [-0.397; 0.607]	0.681	1.000
D	ΔHR≥0	BL	23	0.000 (0.000; 0.000)	13	0.000 (0.000; 0.000)	7	-0.583 [-1.201; 0.036]	-0.499 [-1.029; 0.031]	0.065	0.390
D	ΔHR≥0	AP	23	-0.199 (-0.485; -0.109)	13	-0.045 (-0.389; 0.824)	7	-0.768 [-1.386; -0.149]	-0.658 [-1.188; -0.128]	0.015	0.105
D	ΔHR≥0	OP	23	0.091 (-0.184; 0.412)	12	0.448 (0.106; 1.609)	7	-1.203 [-1.828; -0.577]	-1.031 [-1.567; -0.495]	<0.001	**0.002**
D	ΔHR≥0	FP	19	0.208 (0.016; 1.016)	13	0.878 (0.157; 1.721)	5	-1.027 [-1.670; -0.384]	-0.880 [-1.431; -0.329]	0.002	**0.016**
D	ΔHR≥0	Post	23	0.373 (0.137; 0.632)	13	1.432 (0.089; 3.137)	7	-0.925 [-1.544; -0.307]	-0.793 [-1.323; -0.263]	0.003	**0.027**
D	ΔHR<0	BL	22	0.000 (0.000; 0.000)	25	0.000 (0.000; 0.000)	10	0.372 [-0.206; 0.950]	0.319 [-0.177; 0.814]	0.207	1.000
D	ΔHR<0	AP	22	0.087 (-0.159; 0.588)	25	0.115 (-0.116; 0.423)	10	0.187 [-0.391; 0.765]	0.160 [-0.335; 0.655]	0.527	1.000
D	ΔHR<0	OP	22	0.493 (0.224; 1.179)	25	0.785 (0.067; 1.216)	10	-0.248 [-0.831; 0.334]	-0.213 [-0.712; 0.286]	0.403	1.000
D	ΔHR<0	FP	19	0.877 (0.665; 1.499)	21	0.941 (0.018; 2.043)	8	-0.073 [-0.684; 0.539]	-0.062 [-0.586; 0.462]	0.816	1.000
D	ΔHR<0	Post	22	1.013 (0.463; 1.760)	25	0.866 (0.133; 2.331)	10	0.029 [-0.549; 0.607]	0.025 [-0.470; 0.520]	0.922	1.000
SI	ΔHR≥0	BL	23	0.000 (0.000; 0.000)	13	0.000 (0.000; 0.000)	7	-0.175 [-0.403; 0.052]	-0.402 [-0.924; 0.119]	0.131	0.653
SI	ΔHR≥0	AP	23	-0.376 (-1.017; -0.055)	13	0.089 (-0.403; 0.390)	7	-0.546 [-0.773; -0.318]	-1.251 [-1.773; -0.730]	<0.001	**<0.001**
SI	ΔHR≥0	OP	23	0.086 (-0.130; 0.277)	12	0.526 (0.209; 0.768)	7	-0.242 [-0.473; -0.012]	-0.556 [-1.084; -0.028]	0.039	0.233
SI	ΔHR≥0	FP	19	0.195 (0.053; 0.484)	13	0.617 (0.274; 0.803)	5	-0.269 [-0.506; -0.033]	-0.618 [-1.160; -0.075]	0.026	0.205
SI	ΔHR≥0	Post	23	0.229 (0.063; 0.472)	13	0.475 (0.155; 0.745)	7	-0.249 [-0.476; -0.021]	-0.570 [-1.091; -0.048]	0.032	0.225
SI	ΔHR<0	BL	22	0.000 (0.000; 0.000)	25	0.000 (0.000; 0.000)	10	0.093 [-0.120; 0.306]	0.213 [-0.276; 0.701]	0.393	1.000
SI	ΔHR<0	AP	22	0.037 (-0.158; 0.309)	25	0.058 (-0.087; 0.299)	10	-0.277 [-0.491; -0.064]	-0.636 [-1.125; -0.148]	0.011	0.096
SI	ΔHR<0	OP	22	0.313 (0.174; 0.451)	25	0.365 (0.208; 0.550)	10	0.026 [-0.189; 0.240]	0.059 [-0.433; 0.551]	0.814	1.000
SI	ΔHR<0	FP	19	0.485 (0.387; 0.599)	21	0.491 (0.183; 0.623)	8	-0.001 [-0.227; 0.224]	-0.003 [-0.520; 0.515]	0.992	1.000
SI	ΔHR<0	Post	22	0.499 (0.303; 0.691)	25	0.396 (0.234; 0.696)	10	0.020 [-0.193; 0.233]	0.045 [-0.443; 0.534]	0.856	1.000
LF	ΔHR≥0	BL	23	0.000 (0.000; 0.000)	13	0.000 (0.000; 0.000)	7	-0.755 [-1.436; -0.075]	-0.588 [-1.117; -0.058]	0.030	0.177
LF	ΔHR≥0	AP	23	-0.242 (-0.450; 0.164)	13	-0.051 (-0.250; 2.077)	7	-1.173 [-1.853; -0.493]	-0.913 [-1.442; -0.383]	<0.001	**0.005**
LF	ΔHR≥0	OP	23	0.194 (-0.138; 0.736)	12	0.573 (0.110; 3.239)	7	-1.668 [-2.356; -0.980]	-1.298 [-1.833; -0.762]	<0.001	**<0.001**
LF	ΔHR≥0	FP	19	0.670 (-0.203; 1.043)	13	0.843 (0.271; 4.168)	5	-1.488 [-2.196; -0.781]	-1.158 [-1.708; -0.608]	<0.001	**<0.001**
LF	ΔHR≥0	Post	23	0.179 (-0.196; 0.987)	13	1.537 (-0.032; 4.501)	7	-1.905 [-2.585; -1.224]	-1.482 [-2.011; -0.952]	<0.001	**<0.001**
LF	ΔHR<0	BL	22	0.000 (0.000; 0.000)	25	0.000 (0.000; 0.000)	10	0.499 [-0.137; 1.135]	0.388 [-0.106; 0.883]	0.124	0.496
LF	ΔHR<0	AP	22	0.520 (-0.306; 0.886)	25	0.088 (-0.073; 0.542)	10	0.081 [-0.554; 0.717]	0.063 [-0.431; 0.558]	0.802	0.991
LF	ΔHR<0	OP	22	0.977 (-0.099; 1.668)	25	0.587 (0.072; 1.249)	10	-0.413 [-1.054; 0.227]	-0.322 [-0.820; 0.177]	0.206	0.618
LF	ΔHR<0	FP	19	0.838 (0.228; 1.851)	21	0.696 (0.180; 1.518)	8	-0.234 [-0.907; 0.439]	-0.182 [-0.706; 0.341]	0.495	0.991
LF	ΔHR<0	Post	22	0.580 (-0.154; 1.409)	25	0.642 (0.356; 1.800)	10	-0.650 [-1.286; -0.014]	-0.506 [-1.001; -0.011]	0.045	0.225
HF	ΔHR≥0	BL	23	0.000 (0.000; 0.000)	13	0.000 (0.000; 0.000)	7	-0.616 [-2.231; 1.000]	-0.200 [-0.726; 0.325]	0.455	1.000
HF	ΔHR≥0	AP	23	-0.295 (-0.487; -0.170)	13	-0.147 (-0.301; 0.255)	7	-0.537 [-2.152; 1.079]	-0.175 [-0.700; 0.351]	0.515	1.000
HF	ΔHR≥0	OP	23	-0.022 (-0.279; 0.096)	12	0.712 (0.143; 1.332)	7	-1.545 [-3.179; 0.089]	-0.503 [-1.034; 0.029]	0.064	0.638
HF	ΔHR≥0	FP	19	-0.093 (-0.208; 0.578)	13	0.661 (0.000; 1.581)	5	-1.067 [-2.748; 0.614]	-0.347 [-0.894; 0.200]	0.213	1.000
HF	ΔHR≥0	Post	23	-0.059 (-0.331; 0.395)	13	1.052 (0.209; 1.736)	7	-0.567 [-2.182; 1.049]	-0.184 [-0.710; 0.341]	0.492	1.000
HF	ΔHR<0	BL	22	0.000 (0.000; 0.000)	25	0.000 (0.000; 0.000)	10	0.508 [-1.003; 2.018]	0.165 [-0.326; 0.656]	0.510	1.000
HF	ΔHR<0	AP	22	0.203 (0.022; 1.016)	25	0.121 (-0.000; 0.520)	10	0.586 [-0.924; 2.097]	0.191 [-0.300; 0.682]	0.447	1.000
HF	ΔHR<0	OP	22	0.454 (0.081; 2.256)	25	0.577 (0.030; 0.930)	10	-0.422 [-1.943; 1.099]	-0.137 [-0.632; 0.357]	0.586	1.000
HF	ΔHR<0	FP	19	0.852 (0.238; 2.815)	21	0.811 (-0.004; 1.557)	8	0.056 [-1.542; 1.654]	0.018 [-0.502; 0.538]	0.945	1.000

Metric	HR group	Phase	n SST	SST Median (Q1; Q3)	n WMT	WMT Median (Q1; Q3)	n overlap	Estimate [95% CI]	Std. effect [95% CI]	*p*-value	Holm-adjusted p
HF	ΔHR<0	Post	22	1.474 (0.387; 3.182)	25	0.706 (0.133; 1.592)	10	0.556 [-0.954; 2.067]	0.181 [-0.310; 0.672]	0.470	1.000
LF/HF	ΔHR≥0	BL	23	0.000 (0.000; 0.000)	13	0.000 (0.000; 0.000)	7	-0.089 [-0.430; 0.253]	-0.139 [-0.676; 0.398]	0.612	1.000
LF/HF	ΔHR≥0	AP	23	0.307 (0.018; 0.646)	13	0.448 (0.035; 1.145)	7	-0.326 [-0.668; 0.016]	-0.512 [-1.049; 0.025]	0.062	0.495
LF/HF	ΔHR≥0	OP	23	0.266 (0.063; 0.893)	12	0.125 (-0.194; 0.773)	7	-0.248 [-0.594; 0.098]	-0.389 [-0.933; 0.154]	0.160	1.000
LF/HF	ΔHR≥0	FP	19	0.196 (-0.082; 1.263)	13	0.166 (0.053; 1.023)	5	-0.246 [-0.601; 0.110]	-0.386 [-0.944; 0.172]	0.175	1.000
LF/HF	ΔHR≥0	Post	23	0.209 (-0.107; 0.656)	13	0.142 (-0.164; 0.632)	7	-0.533 [-0.875; -0.191]	-0.838 [-1.375; -0.300]	0.002	**0.022**
LF/HF	ΔHR<0	BL	22	0.000 (0.000; 0.000)	25	0.000 (0.000; 0.000)	10	0.042 [-0.277; 0.362]	0.067 [-0.435; 0.569]	0.795	1.000
LF/HF	ΔHR<0	AP	22	0.003 (-0.391; 0.311)	25	-0.014 (-0.218; 0.351)	10	-0.195 [-0.514; 0.125]	-0.306 [-0.808; 0.196]	0.232	1.000
LF/HF	ΔHR<0	OP	22	-0.058 (-0.454; 0.389)	25	0.232 (-0.282; 0.670)	10	-0.117 [-0.439; 0.205]	-0.184 [-0.689; 0.322]	0.476	1.000
LF/HF	ΔHR<0	FP	19	-0.131 (-0.422; 0.518)	21	0.027 (-0.326; 0.775)	8	-0.115 [-0.452; 0.223]	-0.180 [-0.710; 0.350]	0.505	1.000
LF/HF	ΔHR<0	Post	22	-0.398 (-0.656; 0.139)	25	-0.073 (-0.321; 0.491)	10	-0.402 [-0.722; -0.083]	-0.632 [-1.134; -0.130]	0.014	0.122
